# Development and validation of a bronchoalveolar lavage genomic classifier for acute cellular rejection

**DOI:** 10.1016/j.ebiom.2025.106046

**Published:** 2025-12-02

**Authors:** S. Samuel Weigt, Jin Zhou, Tomoki Okuno, Megan L. Neely, Shahrzad Lari, Vyacheslav Palchevskiy, Jamie L. Todd, Laurie D. Snyder, David Sayah, Michael Y. Shino, John Reynolds, Pali D. Shah, Lianne G. Singer, Marie Budev, Scott Palmer, John A. Belperio

**Affiliations:** a Department of Medicine, David Geffen School of Medicine, University of California, Los Angeles, CA, USA; b Department of Biostatistics, University of California, Los Angeles, CA, USA; c Duke Clinical Research Institute, Durham, NC, USA; d Department of Medicine, Duke University, Durham, NC, USA; e Johns Hopkins Medicine, Baltimore, MD, USA; f University Health Network, University of Toronto, Toronto, Ontario, Canada; g Cleveland Clinic, Cleveland, OH, USA

**Keywords:** Lung transplantation, Acute cellular rejection, Chronic lung allograft dysfunction, Transcriptomics, Genomic classifier

## Abstract

**Background:**

Acute cellular rejection (ACR) is the main risk factor for chronic lung allograft dysfunction (CLAD), but diagnosis requires invasive transbronchial biopsy (TBB). We previously demonstrated the feasibility of bronchoalveolar lavage cell pellet (BAL-cp) gene expression for ACR diagnosis. We sought to develop and validate a genomic classifier for ACR in a multicenter cohort.

**Methods:**

We performed RNA-seq on 806 BAL-cp from 181 lung transplant recipients enrolled in CTOT-20. Differential expression was based on fold difference >2.0 and False Discovery adjusted p-value <0.05. Samples were randomly split 80:20 into training and testing sets. A Random Forest model was optimized for area under the curve (AUC), and the threshold for genomic ACR was selected for classification accuracy. We validated performance in an independent single-centre cohort. Cox models evaluated risk for CLAD.

**Findings:**

From 37 clinically significant ACR and 151 stable control samples, we identified 62 ACR genes, indicating upregulation of T-cell receptor signalling, and downregulation of CTLA4 signalling in cytotoxic lymphocytes, among other enriched pathways. A 31-gene Random Forest model's AUC was 0.99 (SE 0.0053) in the training set, and 0.72 (SE 0.0874) in the test set. At a probability threshold of 0.396, accuracy for distinguishing clinically significant ACR cases from stable controls was 93.1% (specificity 95.4%, sensitivity 83.8%). In the independent validation cohort, accuracy was 82.1% (specificity 87.5%, sensitivity 73.3%). The model classified 138 (17.1%) CTOT-20 samples as genomic ACR. Late genomic ACR (≥90 days posttransplant) associated with increased CLAD risk (HR 2.52, 95% CI 1.47–4.34, p < 0.001).

**Interpretation:**

A BAL-cp genomic classifier can identify ACR, predict CLAD risk, and may be a less invasive alternative to TBB after lung transplant.

**Funding:**

This study was supported by the 10.13039/100000060National Institute of Allergy and Infectious Diseases awards U01AI113315 and U01AI0635594, the 10.13039/100000897Cystic Fibrosis Foundation award PALMER19AB0, and the Saul and Joyce Brandman Foundation Center for Lung Health at UCLA.

**Research in context**

**Evidence before this study**

Acute cellular rejection is the principal risk factor for chronic lung allograft dysfunction. In current practice, acute cellular rejection is diagnosed by transbronchial biopsy, which is invasive and limited by sampling error and subjective interpretation. Prior proof of concept work in a single centre suggested that bronchoalveolar lavage cell pellet gene expression profiling could diagnose acute cellular rejection and predict chronic lung allograft dysfunction.

**Added value of this study**

In a multicenter lung transplant cohort, we demonstrate the utility of bronchoalveolar lavage cell pellet transcriptomics to diagnose acute cellular rejection less invasively than the current standard. Additionally, we show that a genomic classifier is accurate and reproducible across multiple sites and in an independent cohort.

**Implications of all the available evidence**

A genomic classifier that can safely and accurately identify instances of acute cellular rejection missed by transbronchial biopsy provides new opportunities to intervene and potentially prevent the development of chronic lung allograft dysfunction in lung transplant recipients.

## Introduction

Lung transplantation is a life-extending treatment for select patients with advanced lung disease. However, lung recipients experience a higher burden of allograft failure and decreased survival compared to other solid organ transplants. Chronic lung allograft dysfunction (CLAD) is the major factor that limits long-term survival to a median of 6.7 years.^1^ Since there are no effective therapies, the identification and mitigation of CLAD risk factors is critical. The most consistently implicated CLAD risk factor is acute cellular rejection (ACR).^1^, ^2^, ^3^, ^4^, ^5^, ^6^, ^7^ ACR is diagnosed based on pathologist's review of transbronchial biopsies (TBB) and identification of A-grade rejection, which involves perivascular mononuclear infiltrates that may extend to the interstitium and alveolar spaces.^4^ ACR also frequently co-occurs with other pathology patterns, including lymphocytic bronchiolitis (LB), organizing pneumonia (OP), and acute lung injury (ALI).^7^ However, other etiologies commonly implicated with non-A-grade injury patterns include aspiration, infection, and antibody mediated rejection (AMR). This lack of specificity combined with a high incidence of sampling error limits the clinician's ability to reliably diagnose ACR. In a multicenter study, 8% of TBB's yielded an inadequate sample, another 26% are assessed as suboptimal, and interobserver agreement between expert pathologists is poor.^3^ In addition, TBB is further limited by a small but real risk of morbidity.^7^

Bronchoalveolar lavage (BAL) is safer than TBB and samples a larger surface area of lung. In a prior single centre study, we profiled gene expression in the BAL cell pellet (BAL-cp) and showed that A-grade rejection is enriched for immune response genes, including “T-cell receptor signalling” and “natural killer cell mediated cytotoxicity” pathways.^2^ Additionally, a 4-gene genomic classifier for A-grade rejection predicted risk of CLAD. Importantly, some samples with a corresponding TBB negative for A-grade rejection were classified as ACR, suggesting that BAL-cp gene expression may detect ACR missed by TBB.

We hypothesized that BAL-cp gene expression could reliably diagnose ACR and predict risk of CLAD. We leveraged data and BAL-cp collected through the multi-centre Clinical Trials in Organ Transplantation (CTOT)-20 study to determine differential gene expression during ACR and to develop a genomic classifier. Additionally, we used our previously described single centre BAL-cp RNA-seq dataset for independent external validation.

## Methods

### Cohorts and specimen collection

CTOT-20 (NCT02631720) enrolled 803 first, adult lung transplant recipients across five North American Lung Transplant Centres (Duke University, University of California Los Angeles, University of Toronto, Johns Hopkins University, and Cleveland Clinic) in a prospective observational cohort study. Patients were transplanted between December 2015 and August 2018. Patients who completed CTOT-20 were approached for the CTOT extension study (CTOT-ES, NCT04126746) for additional prospective follow-up. BAL fluid samples were collected from each bronchoscopy and processed as previously described.^5^^,^^6^ The studies were approved by each centre's institutional review board (IRB) and all participants provided informed consent.

For this study, we utilized BAL-cp from 200 consecutively transplanted CTOT-20 subjects from 8/28/2016 to 4/15/2017 (UCLA IRB #19–001636). We utilized CTOT-20 and CTOT-ES follow-up data through 7/29/2021.

The CTOT-20 BAL protocol used two 50–60-ml aliquots of isotonic saline instilled into a subsegmental bronchus of the right middle lobe or lingula, consistent with ISHLT guidelines.^5^^,^^6^ Investigators could deviate from the protocol at their discretion based on patient tolerance. Retrieved BAL fluid was pooled and then split into clinical and research specimens with the remaining volume. Research samples were placed on ice and processed within 6 h. BAL fluid was filtered through sterile gauze and cells were separated by centrifugation. Cell pellets were washed and preserved in RNAlater at −80° C (Thermo Fisher Scientific, Waltham, MA, USA).

An independent single centre validation cohort included 219 lung transplant recipients from UCLA who each provided informed consent to participate in a local registry and biorepository, the Lung Transplant Outcomes Study (UCLA IRB #13-000462). Use of this cohort was approved by the principal investigator, John Belperio. Only 1 sample per patient was included and methods for BAL collection, processing, sample selection, and RNA-seq were previously described.^2^

### Definitions and outcomes

Each sample was assigned a clinical phenotype, reflecting the status of the patient including findings on bronchoscopy. The stable control (SC) phenotype refers to samples collected for surveillance with stable lung function, negative TBB (A0, B0, and no ALI or OP), negative microbiology, and no DSA. The clinically significant ACR (csACR) phenotype is defined as mild or greater A-grade rejection (A2, A3, or A4), or minimal A-grade rejection (A1) on a for-cause bronchoscopy. A1 rejection on surveillance bronchoscopy was considered a separate clinical phenotype. A-grade rejection with concurrent positive microbiology or DSA was not included in the csACR phenotype. SC and csACR phenotypes were the outcomes used for differential expression analysis and model development. Additional clinical phenotypes are listed and defined in the Supplemental Material (Table S1).

CLAD was defined as a 20% or greater decline in FEV1 as compared to the average of the two best posttransplant FEV1s, sustained for at least three weeks in the absence of confounders, in keeping with the 2019 ISHLT consensus definition for probable CLAD.^8^ As previously described,^9^ each case meeting PFT criteria for CLAD required adjudication by the enrolling centre to exclude a confounding explanation. CLAD onset was defined as the date of the first threshold FEV1 decline. Patients with a CLAD related death or retransplant, as confirmed by the site investigator, prior to meeting PFT criteria, were also considered as meeting the CLAD endpoint.

### RNA extraction and quality control

Total RNA was extracted with the miRNeasy Kit (Qiagen). Nanodrop (Thermo Fisher Scientific) was used to assess RNA quantity and purity. RNA integrity was assessed by Agilent 2100 (Agilent Technologies, Santa Clara, CA, USA). Samples were advanced to library construction and sequencing only if RNA quantity was greater than 0.2 μg and the RNA integrity number (RIN) was at least 5.8.

### Library construction and sequencing

Messenger RNA was purified from total RNA using poly-T oligo-attached magnetic beads. After fragmentation, the first strand cDNA was synthesized using random hexamer primers, followed by the second strand cDNA synthesis using either dUTP for directional library or dTTP for non-directional library. For the non-directional library, it was ready after end repair, A-tailing, adaptor ligation, size selection, amplification, and purification. The library was checked with Qubit and real-time PCR for quantification and bioanalyzer for size distribution detection. Library preparations were sequenced on an Illumina NovoSeq 6000 sequencing system (paired-end 150-nucleotide read length at >20 million reads per sample).

### Mapping and quantification

Raw data (raw reads) of FASTQ format were firstly processed through fastp. In this step, clean data (clean reads) were obtained by removing reads containing adaptor and poly-N sequences and reads with low quality from raw data. At the same time, Q20, Q30 and GC content of the clean data were calculated. All the downstream analyses were based on the clean data with high quality. Paired-end clean reads were aligned to the reference genome (NCBI/UCSC/Ensembl) using the Spliced Transcripts Alignment to a Reference (STAR) software, which uses sequential maximum mappable seed search in uncompressed suffix arrays followed by seed clustering and stitching procedure. FeatureCounts was used to count the read numbers mapped of each gene, and then Reads Per Kilobase of exon model per Million mapped reads (RPKM) of each gene was calculated based on the length of the gene and reads count mapped to this gene.

### Differential expression analysis

Differentially expressed genes during csACR vs. SC samples were detected using *LIMMA and voom* packages.^10^ We only included genes with >2 mean reads per million mapped reads. LIMMA was used in conjunction with voom, which weighs the mean–variance relationship of the log-counts needed for accurate generalized linear modelling. Empirical Bayes estimator was adopted,^10^ with adjustment for age, self-reported sex, days to sample collection (log2 transformed), transplant type (bilateral or single), primary graft dysfunction grade 3 at 72 h, and use of induction. Correlation between repeated measures were considered and adjusted in differential expression analysis. Candidate genes were identified based on an absolute fold change >2.0 and Benjamini−Hochberg adjusted p-value less than 0.05.

### Enrichment and pathway analyses

We used Ingenuity Pathway Analyses (IPA) software to examine the biologic relevance of the list of differentially expressed genes.

### Classifier modelling

CTOT-20 samples were randomly split 80:20 into training and test sets (other proportions were also explored without major differences). Models were trained to distinguish csACR from SC. Differentially expressed genes were included as features for classifier modelling. For modelling, we treated repeated measures as independent observations. Several machine learning methods were evaluated, including simple logistic regression, Elastic-Net with 10-fold Cross-Validation, and Random Forest (RF) with 10-fold Cross-Validation. We selected a RF model as they are generally robust to overfitting and capable of learning non-linear relationships. We tuned the number of variables available for splitting at each tree node (mtry range 1–15) and the number of trees (ntree range 100–5000) optimized for area under the curve (AUC). We used the Boruta variable selection method,^11^ an all-relevant feature selection method based on Random Forest, to identify a robust set of features. This method generates shadow features and compares the Z-score of the Mean Decrease Accuracy of all original features against the maximum Z-score of the shadow features over 1000 iterative runs. This process categorizes features into Confirmed or Rejected sets, effectively eliminating bias and ensuring only statistically relevant features are retained for modelling.

The final locked model threshold was selected based on optimal accuracy for distinguishing csACR from SC in the training set and validated in the test set. Classification performance was reported as sensitivity, specificity, and accuracy, as well as negative predictive value (NPV) and positive predictive value (PPV) where appropriate. We applied the classifier to the entire dataset and describe the proportion of non-csACR phenotypes classified as genomic ACR (gACR).

### CLAD survival analyses

We used Cox models to determine risk factors for CLAD. Time was calculated from day 90 posttransplant to either CLAD onset or censor date (last PFT). Covariates were selected based on modelling and findings from the full CTOT-20 cohort.^12^ Time-independent covariates included baseline characteristics and “early” events occurring up to 90 days post-transplant, including gACR. “Late” events occurring after 90 days was considered time-dependent covariates. Class I and class II DSA were included in models only if occurring >90 days (not early DSA), as these events more reflect humoural responses that persist after transplant.

Analyses were conducted using the Bioconductor suite of packages in the R statistical software environment version 4.2.1. Packages “randomForest” and “glmnet” were used for classifier modelling.

### Role of funders

The funders of the study had no role in the study design, data collection, data analysis, data interpretation, or writing of the report. The Clinical Trials in Organ Transplant Publications Subcommittee reviewed and approved this article submission. No pharmaceutical company or other agency paid authors to submit this article.

## Results

**Fig. 1:**
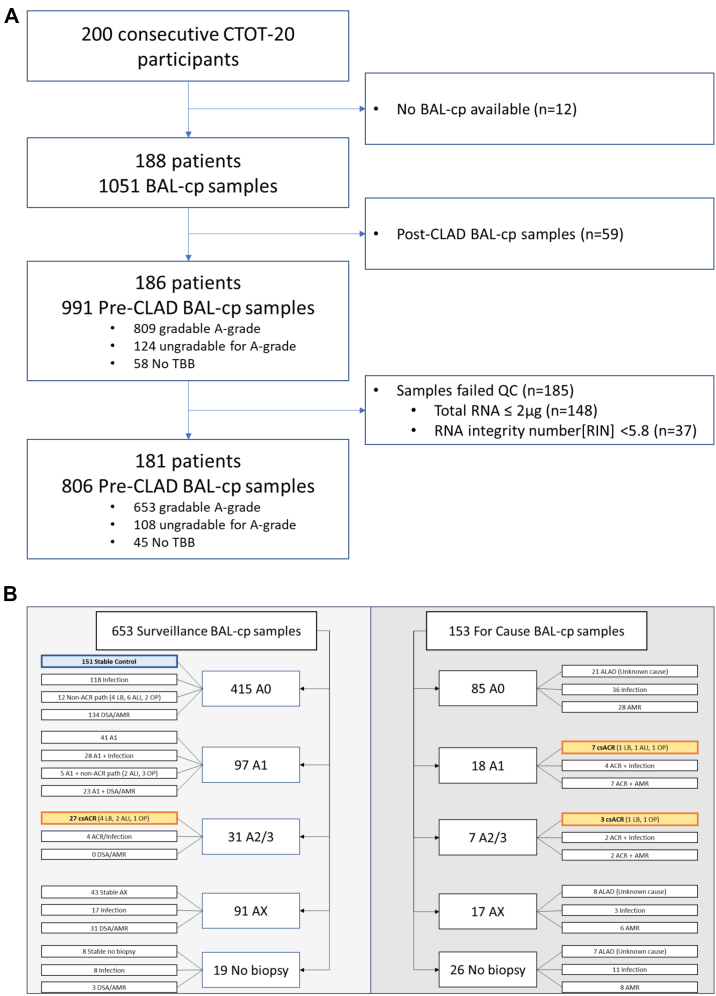
(A) Consort diagram for inclusion of patients and samples. (B) Distribution of A-grades by surveillance and for cause biopsies, and also indicating SC (highlighted blue) and csACR (highlighted orange) samples.

Of the 200 consecutively enrolled CTOT-20 patients screened, 186 had at least 1 pre-CLAD BAL-cp available (Fig. 1). Of the 991 pre-CLAD BAL-cp, 185 (18.6%) failed QC, most (148) for insufficient RNA quantity. The characteristics for passed and failed QC are shown in Table S2. The main characteristic associated with insufficient RNA quantity was the volume of BAL fluid submitted for research (Figure S1). Volumes below 10 ml frequently yielded insufficient RNA quantity, while volumes above 20 ml yielded sufficient RNA quantity more than 90% of the time. After exclusion of BAL-cp that failed QC, 181 patients with 806 samples remained eligible.

An independent single centre (UCLA) cohort including 219 lung transplant recipients served as external validation. The subject inclusion and sample selection criteria were previously described.^2^ As compared to CTOT-20, the external cohort was more racially and ethnically diverse and had more single lung recipients (Table 1). All patients in the UCLA cohort received induction, while some patients in CTOT-20 received no induction based on centre specific practices. CTOT-20 subjects had between 1 and 11 BAL-cp (median = 5). The UCLA external cohort only included 1 BAL-cp per patient.

**Table 1:** Characteristics of included subjects.

Variable	Level	CTOT cohort N (%) N (%) = 181	UCLA cohort N (%) = 219
Sex	F	80 (44.2)	83 (37.9)
	M	101 (55.8)	136 (62.1)
Age at Transplant, years AGETRPL	Mean (SD)	55.2 (13.3)	59.7 (11.2)
	Median (IQR)	59 (51–64)	62 (56–67)
Race	American Indian or Alaska Native	1 (0.5)	0
	Asian	4 (2.2)	7 (3.2)
	Black or African American	4 (2.2)	8 (3.7)
	Unknown or Not Reported	5 (2.8)	38 (17.4)
	White	167 (92.3)	166 (75.8)
Ethnicity	Hispanic or Latino	8 (4.4)	32 (14.6)
	Not Hispanic or Latino	169 (93.4)	187 (85.4)
	Not Reported	2 (1.1)	0 (0)
	Unknown	2 (1.1)	0 (0)
UNOS native lung category	A (obstructive lung disease)	61 (33.7)	58 (26.5)
	B (pulmonary vascular disease)	6 (3.3)	19 (8.7)
	C (cystic fibrosis)	28 (15.5)	12 (5.5)
	D (restrictive lung disease)	86 (47.5)	130 (59.4)
Transplant type	Bilateral	141 (77.9)	118 (53.9)
	Single	40 (22.1)	101 (46.1)
LAS at transplant	Mean (SD)	42.9 (14.4)	48.0 (16.1)
	Median (IQR)	38.0 (33.7–45.9)	43.2 (35.9–55.5)
HLA Mismatch^a^	1–3	28 (15.5)	15 (13.5)
	4–6	153 (84.5)	96 (86.5)
Induction med	None	98 (54.1)	0
	Basiliximab	73 (40.3)	105 (47.9)
	ATG	10 (5.5)	114 (52.1)
PGD3 within 72 h	Yes	26 (14.4)	40 (18.3)
	No	155 (85.6)	179 (81.7)
Follow up time, days	Mean (SD)	1298 (464)	1722 (906)
	Median (IQR)	1555 (999–1640)	1568 (1030–2282)
BAL cp samples per patient that passed QC	Mean (SD)	4.5 (2.5)	1 (0.1)
	Median (IQR)	4 (2–6)	1 (1–1)

a HLA data unavailable from 108 patients in UCLA cohort.

### BAL-cp phenotypes

In the CTOT-20 cohort, 56 (6.9%) pre-CLAD BAL-cp had an A-grade of A2 or greater or symptomatic A1 on a simultaneously collected TBB. Of these 56, 9 met criteria for possible or probable AMR, 6 met criteria for RVI, and 4 met criteria for bacterial infection. Therefore, 37 (4.6%) samples met criteria for the csACR phenotype used for differential expression analyses and classifier training. Another 151 samples (18.7%) met criteria for the SC phenotype. The full list and counts of phenotypes for CTOT-20 and external cohorts can be found in the supplemental material (Table S1).

### Differential gene expression, functional annotation, and pathway enrichment

**Fig. 2:**
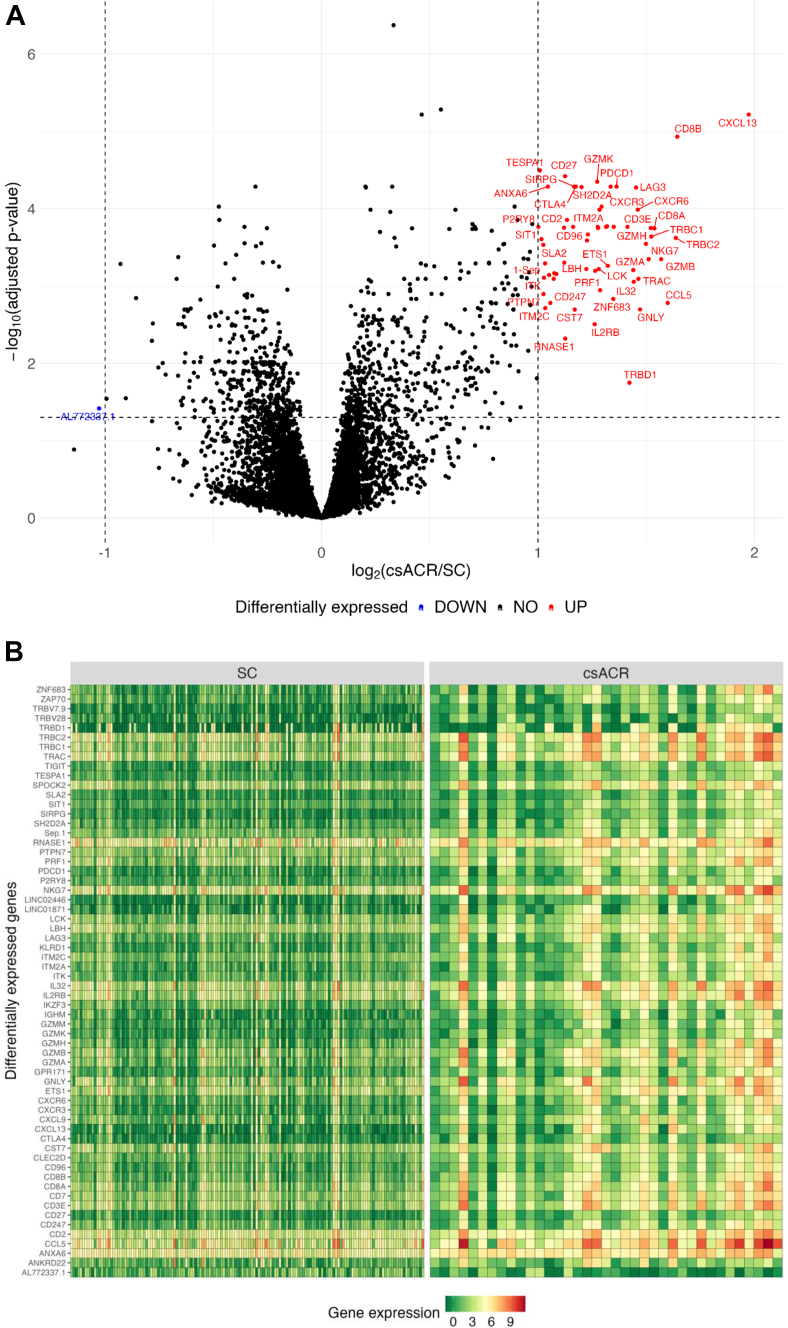
Differential expression analysis of bronchoalveolar lavage cell pellet from lung transplant recipients with clinically significant ACR (csACR). Upregulated or downregulated transcripts during csACR with a False Discovery Rate (FDR) adjusted P-value <0.05 and a log2 fold change >1 or < −1 are shown by volcano plot (A) and heatmap (B).

After filtering out transcripts with low read count across samples, 10,878 out of 58,735 transcripts remained. Differential expression analysis comparing csACR with SC identified 62 genes, 61 of which were upregulated during csACR (Fig. 2, Table 2).

**Table 2:** Differentially expressed genes (n = 62) during csACR in BAL cell pellet (Benjamini-Hochberg corrected p-value <0.05, and absolute FC ≥ 2.0).

gene_name	logFC	adj.p-value	gene_name	logFC	adj.p-value	gene_name	logFC	adj.p-value
*CXCL13*^a^	1.97	0.00001	SH2D2A	1.34	0.00005	CD2^a^	1.13	0.00014
CD8B^a^	1.64	0.00001	ETS1^a^	1.32	0.00055	RNASE1	1.13	0.00479
TRBC2	1.64	0.00024	IGHM	1.32	0.00017	CD27^a^	1.13	0.00004
CCL5^a^	1.60	0.00166	LINC02446	1.31	0.00017	LBH	1.12	0.00050
GZMB	1.57	0.00045	CXCR3^a^	1.29	0.00009	CLEC2D^a^	1.12	0.00018
CD8A	1.54	0.00018	PRF1	1.29	0.00113	TRBV28	1.08	0.00070
TRBC1^a^	1.52	0.00023	ITM2A^a^	1.28	0.00010	ZAP70	1.08	0.00068
CD3E^a^	1.52	0.00018	LCK^a^	1.28	0.00060	ANKRD22	1.07	0.00081
NKG7	1.51	0.00045	CXCL9	1.28	0.00018	CD247^a^	1.06	0.00166
GZMH	1.50	0.00029	KLRD1^a^	1.28	0.00017	TRBV7-9^a^	1.05	0.00072
GNLY	1.47	0.00201	GZMK^a^	1.27	0.00004	ANXA6^a^	1.05	0.00005
TRAC^a^	1.46	0.00081	SPOCK2^a^	1.26	0.00064	ITM2C	1.03	0.00193
CXCR6^a^	1.46	0.00010	IL2RB	1.26	0.00312	SEPTIN1^a^	1.03	0.00051
LAG3	1.45	0.00005	GZMM^a^	1.23	0.00022	ITK	1.03	0.00078
IL32	1.44	0.00088	CD96^a^	1.23	0.00026	AL772337.1^a^	−1.03	0.03830
GZMA	1.44	0.00062	CD7	1.22	0.00060	PTPN7	1.03	0.00127
TRBD1	1.42	0.01786	TIGIT^a^	1.20	0.00005	SLA2	1.02	0.00029
LINC01871	1.41	0.00017	CTLA4	1.17	0.00005	SIT1	1.02	0.00025
PDCD1^a^	1.36	0.00005	CST7^a^	1.17	0.00202	TESPA1^a^	1.01	0.00003
IKZF3^a^	1.35	0.00017	SIRPG^a^	1.17	0.00005	P2RY8	1.00	0.00017
ZNF683	1.35	0.00147	GPR171^a^	1.16	0.00017			

a Indicates transcript is included in 31-gene classifier model.

**Fig. 3:**
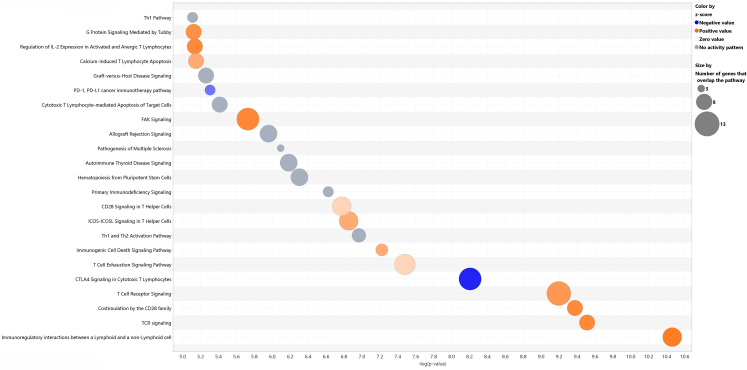
Top canonical pathways enriched in 62-gene ACR differential expression list.

Among the 62 differentially expressed genes, Immunoregulatory interactions between a lymphoid and non-lymphoid cell (z-score = 3.162, Fisher's exact p = 3.45e-11) and TCR signalling (z-score = 2.828, Fisher's exact p = 3.08e-10) were the top-two activated pathways with csACR, while CTLA4 signalling in cytotoxic lymphocytes (z-score = −2.111, Fisher's exact p = 6.21e-09) and PD-1, PD-L1 cancer immunotherapy (z-score = −1.342, Fisher's exact p = 4.94e-06) were the top negatively regulated pathways (Fig. 3). We also examined predicted upstream regulators based on the 62 gene list, and the top upstream regulator was IL-2, followed by pembrolizumab, a checkpoint inhibitor that blocks PD-1 (Table S3).

### ACR classifier development and diagnostic performance

All 62 differentially expressed genes were utilized for classification modelling. In the RF model with mtry = 1 and ntree = 700, 10 training set subsets yielded an average AUC 0.88 (SE = 0.0393). In the training set the AUC was 0.99 (SE = 0.0051), and in the test set the AUC was 0.70 (SE = 0.0931). In order to reduce the number of genes required for classification, we applied the Boruta variable selection method. This reduced the model to 31 genes with almost identical performance to the 62-gene model, where 10 training set subsets yielded an average AUC 0.90 (SE = 0.0334), the entire training set AUC was 0.99 (SE = 0.0053), and the test set AUC was 0.72 (SE = 0.0874) (Table 3). The relative importance of each gene in the model is shown in Figure S2.

**Table 3:** Performance characteristics of BAL-cp 31 gene genomic classifier for differentiating clinically significant ACR and stable control biopsies across training set, test set, CTOT-20 centre, and external cohorts.

	n	AUC	SE	95% CI	Spec	Sens	Accuracy
Average of 10 validation sets	15∗10	0.90	0.0335	–	–	–	–
Training set (80% of CTOT-20)	150	0.99	0.0053	0.98–1.00	–	–	–
Test set (20% of CTOT-20)	38	0.72	0.0874	0.53–0.88	–	–	–
CTOT-20 Total	188	0.96	0.0155	0.92–0.98	95.4%	83.8%	93.1%
CCF/JH/UCLA	62	0.93	0.0312	0.87–0.99	95.6%	70.6%	88.7%
DUMC	63	0.98	0.0157	0.94–1.00	96.2%	90.0%	95.2%
Toronto General Hospital	63	0.99	0.0124	0.95–1.00	94.3%	100.0%	95.2%
UCLA Single Center Cohort	78	0.90	0.0340	0.83–0.96	87.5%	73.3%	82.1%

Optimizing for accuracy in classifying csACR and SC samples, we locked the final 31-gene model at a predicted probability threshold of 0.396. We interpret the results dichotomously as “positive” for gACR if the probability exceeded 0.396, or “negative” if less than or equal to 0.396. In the CTOT-20 cohort, the classifier distinguished csACR from SC samples with 93.1% accuracy (specificity 95.4%, sensitivity 83.8%). The performance was stable across CTOT-20 centres (Table 3) and surveillance windows (Figure S3 and Table S4). In the external cohort,^2^ 26 of the 31 candidate genes were available after filtering. This classifier distinguished csACR from SC with 82.1% accuracy (specificity 87.5%, sensitivity 73.3%).

We also evaluated the classifier's performance for differentiating broader definitions of ACR from other phenotypes with a gradable TBB (Table S5). The best classification performance was for A-grade ≥ A2 in surveillance samples after excluding infections (Accuracy 88.5%, NPV 97.9%). The full performance characteristics under surveillance and for cause indications for various definitions of ACR are shown in Table S5.

TBB diagnosed A-grade rejection ≥ A1 in 153 instances, including 25 (19.7%) out of 127 for cause TBB and 128 (20.2%) out of 542 surveillance TBB (p = 1.0; Fisher's exact test). TBB diagnosed A-grade rejection ≥ A2 in 7 (5.5%) for cause TBB and 31 (4.9%) surveillance TBB (p = 0.82; Fisher's Exact test). By comparison, the classifier predicted gACR in 138 (17.1%) instances, including 22 (20.4%) of the 108 samples where TBB was ungradable for A-grade rejection and 13 (28.9%) of the 45 samples where TBB was not performed (Table S6). In contrast to A-grades from TBB, the incidence of gACR was significantly higher in for cause than in surveillance bronchoscopies (38/153, 24.8% vs. 99/650, 15.2%, p = 0.006; Fisher's Exact test). Among the 3 samples where surveillance or for cause indication was not recorded, one was classified as gACR.

**Fig. 4:**
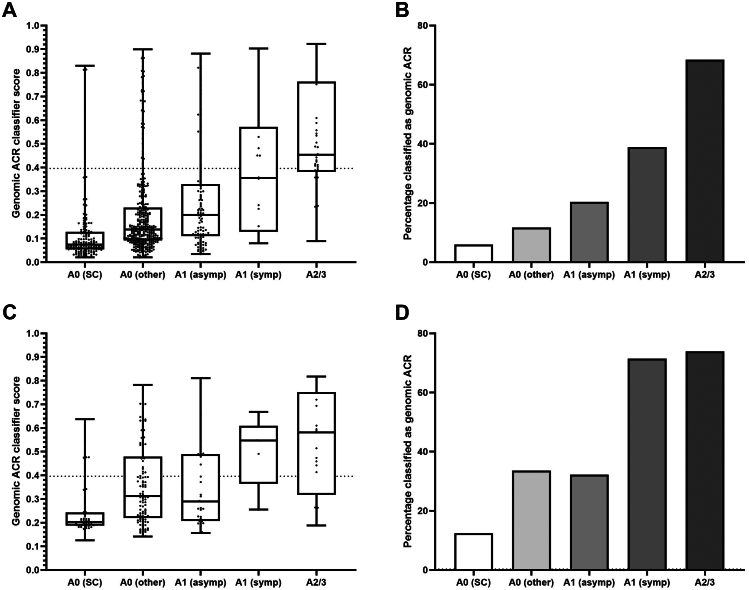
Genomic ACR classifier scores and percentage classified as genomic ACR increase with increasing A-grade in both the CTOT-20 (A & B) and the UCLA cohorts (C & D).

The classifier model's predicted probability of ACR increased with increasing A-grade (Fig. 4). Among samples with injury patterns other than A-grade rejection, 33.3% (3/9) of LB, 17.4% (4/23) of OP, and 21.7% (5/23) of ALI were classified as gACR (Figure S4). Similarly, in the absence of A-grade rejection, a minority of infection samples were classified as gACR (Figure S5). Only 10.5% of de novo DSA samples and 19.4% of AMR samples (probable subclinical or clinical) were classified as gACR, but when csACR occurred concurrent with de novo DSA, the proportion classified as gACR increased to 44.4% (Figure S6).

To explore inter and intrasubject differences in gACR performance, we compared the concordance of the gACR test with A-grade (A0 vs. ≥A1), excluding AX biopsies. The concordance of gACR with A-grade was 77.4%. We then calculated the average concordance within each subject and averaged this intrasubject concordance for all subjects. The average intrasubject concordance was 80.7%, suggesting that within a given subject, the classifier performance is similar to the overall performance where subject is ignored. To visualize classifier behaviour over time, for each of the 143 patients who contributed more than 1 sample we created longitudinal plots of gACR scores, labelled for injury events, infections, augmented immune suppression, and CLAD (Figure S7). Some patterns are appreciated. There are multiple subjects who spike their gACR score concurrent with A2 rejection, then recover to baseline expression levels (e.g. 71,101,057, 71,101,087, 71,119,011, 71,121,040, 79,939,078, and 79,939,090), while others remain persistently elevated or quickly rebound (71,101,071, 71,121,086, 71,121,097, and 71,139,103). Another recognizable pattern is where gACR scores spike while TBB is negative and in some of these cases the gACR scores recover to normal (e.g. 71,101,082, 71,121,062, 71,123,074, 71,139,072, and 71,139,089), while other cases remain persistently elevated (e.g. 71,101,102, 71,139,063, and 71,139,093). These may represent missed opportunities to intervene and minimize lung allograft damage.

### Genomic ACR and CLAD

Among the 181 CTOT-20 patients, 178 were assessable for CLAD, of which 62 met our definition for CLAD (55 by PFT criteria and 7 by CLAD-related death or re-transplantation). Factors associated with CLAD in univariable analysis included the late occurrence of class II DSA (HR 2.25, 95% CI 1.25–4.04, p = 0.007; Cox model), late ALI (HR 3.96, 95% CI 1.80–8.68, p < 0.001; Cox model), and late OP (HR 2.25, 95% CI 1.18–4.30, p = 0.014; Cox model) (Table 4). Additionally, the late occurrence of ACR by various definitions were each associated with CLAD risk, including AR or LB (A1 or greater A-grade or B1R or greater B-grade) (HR 2.04, 95% CI 1.21–3.46, p = 0.008; Cox model), csACR (HR 2.06, 95% CI 1.05–4.03, p = 0.036; Cox model), and gACR (HR 2.52, 95% CI 1.47–4.34, p < 0.001; Cox model). In the external cohort, gACR was also associated with CLAD in a univariable Cox model (HR 1.58, 95% CI 1.07–2.34, p = 0.022; Cox model).

**Table 4:** Univariable associations between covariates of interest and probable CLAD.

Covariate of interest^a^	Probable CLAD	
	Effect estimate HR (95% CI)	p-value
Age at transplant (continuous, per 5 yrs)	0.99 (0.97–1.00)	0.120
Sex (male vs. female)	0.69 (0.42–1.15)	0.158
Race (white vs. not)	1.01 (0.40–2.53)	0.980
UNOS native lung disease category		
B, C, D vs. A	0.99 (0.58–1.69)	0.976
LAS at transplant (continuous, per 10 points)	1.00 (0.98–1.02)	0.859
Transplant type (single vs. bilateral)	1.41 (0.80–2.51)	0.238
Total HLA mismatches (4–6 vs. 1–3)	1.17 (0.57–2.37)	0.669
PGD grade 3 within 72 h (yes vs. no)	1.39 (0.72–2.67)	0.330
Early ALI	0.46 (0.20–1.05)	0.064
**Late ALI**	**3.96 (1.80–8.68)**	**<0.001**
Early OP	0.36 (0.11–1.19)	0.094
**Late OP**	**2.25 (1.18–4.30)**	**0.014**
Early AR or LB	0.83 (0.49–1.41)	0.494
**Late AR or LB**	**2.04 (1.21–3.46)**	**0.008**
Early clinically significant ACR	0.96 (0.46–1.2.03)	0.919
**Late clinically significant ACR**	**2.06 (1.05–4.03)**	**0.036**
Early genomic ACR	0.63 (0.31–1.29)	0.207
**Late genomic ACR**	**2.52 (1.47**–**4.34)**	**<0.001**
Early pseudomonas aeruginosa	0.70 (0.31–1.59)	0.397
Late pseudomonas aeruginosa	1.10 (0.52–2.33)	0.795
Early respiratory viral infection	1.68 (0.89–3.15)	0.109
Late respiratory viral infection	1.16 (0.66–2.03)	0.614
Aspergillus fumigatus^b^	0.96 (0.41–2.23)	0.915
CMV infection^b^	1.48 (0.86–2.56)	0.157
DSA class I^c^	1.78 (0.76–4.18)	0.185
**DSA class II**^c^	**2.25 (1.25–4.04)**	**0.007**

CLAD, chronic lung allograft dysfunction; CMV, cytomegalovirus; DSA, donor specific antibody; HLA, human leukocyte antigen; LAS, lung allocation score; PGD, primary graft dysfunction; TLC, total lung capacity; UNOS, United Network for Organ Sharing.

a For variables described as early or late, early was defined as occurring ≤90 days posttransplant, late was defined as occurring >90 days posttransplant.

b For CMV and Aspergillus Fumigatus infections, early events were very rare, so posttransplant events were combined into a single time-dependent covariate.

c Detected >90 days posttransplant.

In order to determine whether early and late gACR are risk factors for CLAD while controlling for potential confounders, we adjusted for site, transplant type (single vs. bilateral), class I and class II DSA, and CMV infection. We did not adjust for A-grade ACR, LB, OP, or ALI as these biopsy findings may be mediators between gACR and CLAD. We modelled early and late gACR separately, and found that late gACR (adjusted HR 2.02, 95% CI 1.13–3.61, p = 0.02; Cox model), but not early gACR (adjusted HR 0.69, CI 0.33–1.43, p = 0.32; Cox Model) is associated with CLAD.

### Persistent genomic ACR

**Fig. 5:**
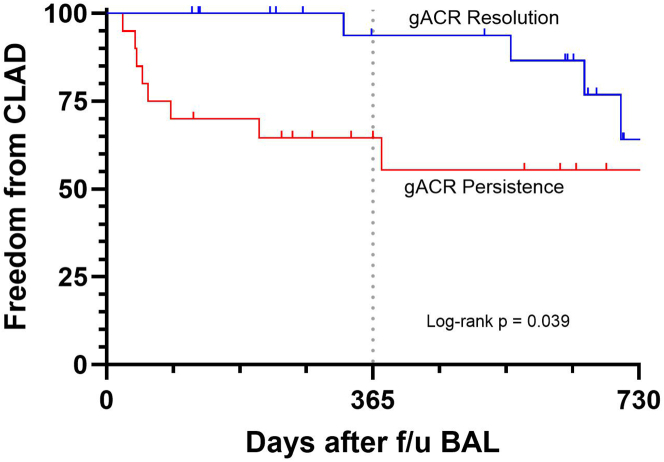
Freedom from CLAD among patients with late gACR, stratified by resolution vs. persistence of gACR, where vertical dotted line indicates 1-year after follow-up BAL where 35.4% of gACR persistence cases and 6.3% of gACR resolution cases had developed CLAD.

Among 56 instances of first late gACR, 43 cases had at least one follow-up sample available, including 22 instances where gACR resolved and 21 where gACR persisted on follow-up BAL. Treatment with augmented immune suppression at the first instance of late gACR was associated with greater resolution of gACR on follow-up (13/18 treated vs. 9/25 untreated, p = 0.03; Fisher's exact test). Persistence of late gACR on follow-up BAL was associated with significantly reduced freedom from CLAD (p = 0.039; log-rank test) (Fig. 5). Among cases with resolution of late gACR, only 6.3% (one subject) developed CLAD within one year, while 35.4% of persistent late gACR cases developed CLAD within 1 year.

### Comparison with other lung allograft rejection gene expression signatures

We compared our BAL-cp gACR classifier to our previously described 4-gene classifier,^2^ as well as to other described signatures of rejection; the 11-gene common rejection module,^13^ the CLAD airway transcriptome classifier^14^ and the LB metagene.^15^ We used the same 80:20 split and seed used for the derivation of our classifier, as well as the same mtry and ntree ranges for RF modelling. Based on AUC, each signature showed good performance in at least one data set (training, test, or external), but only gACR performed well across all 3 (Table 5). Within the external UCLA cohort, the 31-gene gACR classifier performed better than each comparator gene list (DeLong test).

**Table 5:** Performance for different models with the same 80:20 split ratio and seed for the Random Forest approach.

	Tuning parameters		# of genes			AUC		
	mtry	ntree	Original model	CTOT-20 dataset	UCLA dataset	CTOT-20 training set	CTOT-20 test set	UCLA validation (DeLong P)
31-gene genomic ACR classifier	1	700	31	31	26	0.99	0.72	0.89 (reference)
4-gene ACR classifier	1	400	4	4	4	0.64	0.68	0.79 (0.159)
Common rejection module	11	400	11	11	11	0.70	0.73	0.71 (0.022)
CLAD airway transcriptome classifier	2	1800	25	24	20	0.78	0.63	0.61 (0.001)
LB Metagene	3	900	61	61	61	0.74	0.85	0.53 (<0.001)

## Discussion

In a previous single centre proof-of-concept study, we demonstrated that BAL-cp gene expression could identify ACR after lung transplantation,^2^ building upon smaller studies that showed BAL-cp gene expression can provide insight to mechanisms of ACR and CLAD.^16^, ^17^, ^18^, ^19^ In this multicenter study, we aimed to refine and validate a gACR classifier. We identified 62 differentially expressed genes during ACR, from which we developed our 31-gene gACR classifier that performs well across all CTOT-20 sites, and in an independent cohort. The serial sample collection in CTOT-20 also allowed us to demonstrate stable classifier performance over time. Similar to A-grade ACR, we show that gACR occurring after 90 days is associated with increased CLAD risk, providing construct validation.

The objective of surveillance bronchoscopy protocols employed by most transplant centres^20^ is to identify ACR early, where treatment may reverse injury and prevent progressive allograft dysfunction. While we lack evidence that surveillance bronchoscopy prevents CLAD,^21^, ^22^, ^23^, ^24^ the rationale is based on this theoretical benefit.^20^ However, surveillance bronchoscopy relies on the quality of TBB and the pathologist interpreting it. As a part of CTOT-20, pathologists from each site participated in a working group to harmonize definitions of histopathologic findings and the terminology used in reports, although there was no central read. In this study, 20.1% of samples with a paired biopsy were diagnosed with A-grade ACR (153 total episodes), which is similar to prior reports^25^, ^26^, ^27^ and to the full CTOT-20 cohort.^28^ Multiple studies show that TBB is limited by frequent suboptimal or inadequate tissue sampling, subjective interpretation with high interobserver disagreement,^3^^,^^29^^,^^30^ and risk of morbidity.^31^ Furthermore, heterogeneous pathology may be missed due to the relatively small area of lung sampled by TBB. The inability for surveillance TBB to consistently detect ACR may hamper efforts to prevent CLAD.

Molecular technologies have the potential to improve diagnostic accuracy and safety. In one TBB microarray study, a T-cell mediated rejection (TCMR) archetype correlated with histologic TCMR (A-grade ACR ≥ A1).^32^ The same group also showed that gene expression in mucosal biopsies from the third bronchial bifurcation could detect rejection.^33^ Gene expression from airway brushings may also identify ACR and CLAD.^14^^,^^15^ While these approaches appear promising, the potential for sampling error still exists. Paired TBBs from the same bronchoscopy exhibit variance in gene expression, and the authors concluded that two to three pieces from each bronchoscopy would probably be needed to offset this variance.^32^

BAL samples a larger area of lung than TBB, airway biopsies, or airway brushings, potentially eliminating sampling error. However, utility depends on adequacy of RNA. We excluded less than 1 in 5 BAL-cp based on prespecified thresholds for RNA quantity and quality. A simple protocol modification that sets aside at least 20 ml of BAL fluid would virtually eliminate the RNA quantity issue. An additional small percentage of RNA samples were too degraded for RNA-Seq. However, our requirement for RIN ≥5.8 was conservative. The previously sequenced UCLA cohort included 14 samples (∼6%) with RIN between 5.8 and 3.4. The classifier performed well despite the inclusion of more degraded RNA. Of the 40 CTOT-20 samples excluded for low RIN, 24 had RINs between 3.4 and 5.8. Therefore, tolerance of RIN as low as 3.4, along with volumes of at least 20 ml, should allow assessment of nearly all BAL-cp.

Our 62-gene list of differentially expressed genes during csACR provides important mechanistic insights. “T-cell receptor signalling” was among the top canonical pathways, replicating our prior study.^2^ T-cell activation occurs via T-cell receptor (TCR)-major histocompatibility complex (MHC) plus antigenic peptide complex (Signal 1) along with positive co-stimulation through T-cell based CD28 interacting with B7 ligands on antigen presenting cells (Signal 2). Predictably, “Costimulation by the CD28 family” was also a top activated pathway. Activated Type 1 T-helper (Th1) cells produce interleukin (IL)-2, among other cytokines, and are responsible for cell-mediated immunity, including ACR. The “Th1 Pathway” was significantly enriched, the IL-2 receptor subunit IL2RB transcript was increased, and IL-2 was a top predicted upstream regulator. During ACR, activated cytotoxic T-lymphocytes (CTL) migrate to sites of immune activation and induce the death of targeted foreign cells. Cytotoxic T-lymphocyte-associated protein 4 (CTLA-4) functions to down-regulate the CTL response and protect against maladaptive cytotoxicity. Similarly, activated PD-1 limits TCR signaling and thereby inhibits T-cell activation, proliferation and cytokine production. It is noteworthy that both CTLA4 and PDCD1 gene transcripts were significantly increased during csACR, indicating attempts to limit the allo-response. However, both the “CTLA-4 signaling in Cytotoxic T-lymphocytes” and the “PD-1, PD-L1 cancer immunotherapy” pathways appear paradoxically inactivated during csACR; the negative z-scores indicate downstream messaging and effector proteins are not inhibited as would be expected. This suggests that the usual brakes in the system are overwhelmed by the alloimmune response. As a consequence, pathways like “Immunogenic cell death signaling” and “Cytotoxic T-lymphocyte mediated apoptosis of target cells” are upregulated, as are the transcripts for perforin 1 and multiple granzyme genes, which encode for the proteins that carry out the death-inducing functions of granule exocytosis used by CTLs.

The single most upregulated gene during csACR was CXCL13, similar to our prior study.^2^ CXCL13 interacts with CXCR5 on B-lymphocytes and T follicular helper (Tfh) cells, which are essential for germinal centre formation, affinity maturation, and the development of most high affinity antibodies and memory B-lymphocytes. While this finding is somewhat unexpected with acute “cellular” rejection, B-lymphocytes are well known to participate in cellular immunity through antigen presentation and release of cytokines and chemokines. Thus, a CXCL13 driven memory B-cell response, inherently resistant to steroid treatment, could propagate allorecognition and allograft destruction, manifesting as CLAD.

AL772337.1 was the transcript carrying the greatest weight (importance) and the only downregulated transcript in our classifier model. AL772337.1 is a long non-coding (lnc) RNA with no known function and no known role in ACR. lncRNAs often regulate transcription of immune-related genes via chromatin remodelling, transcriptional interference, or sequestration of miRNA preventing them from silencing their target genes. More work is required to determine the role of AL772337.1 in lung allograft rejection.

Compared with other molecular classifiers in transplantation, our classifier performs favourably. In the CARGO heart transplant cohort, Allomap® (CareDx) had an AUC 0.71 (95% CI 0.54 to 0.84). As a screening test for rejection,^34^ a score of 35 has NPV of 99%, while the PPV is comparatively low.^35^ Donor derived cell free DNA (dd-cfDNA) in blood indicates allograft injury and is available for kidney, heart, and lung transplant surveillance. When used as surveillance for acute lung allograft dysfunction, the estimated sensitivity of dd-cfDNA was 74%, specificity of 88%, positive predictive value of 43% and negative predictive value of 97%.^36^ However, dd-cfDNA may not distinguish ACR from other causes of lung injury. A positive dd-cfDNA test still requires bronchoscopy with TBB. Our BAL-cp genomic classifier is less invasive than TBB, and BAL can simultaneously rule out infection. Given that TBB misses some ACR, the PPV of gACR may be falsely low. Importantly, gACR was approximately 4 times more prevalent than csACR. Although the classifier performance for differentiating csACR from SC was stable over time, only late gACR (≥90 days posttransplant) was associated with future CLAD risk. This is consistent with TBB diagnosed ACR in our study, in the full CTOT-20 cohort,^12^ and from other reports.^37^^,^^38^ Persistence of late gACR in follow-up BALcp sample was also associated with earlier CLAD development than late gACR that resolves, suggesting serial testing may be useful to follow treatment responses.

There are several limitations of our study. We acknowledge the apparent contradiction of using A-grade rejection as the “ground truth” for ACR modelling after pointing out the shortcomings of TBB. Importantly, our model was trained to differentiate csACR from SC. As we define it, csACR is likely specific for ACR, minimizing potential confounders to gene expression. Our equally stringent definition for SC is almost certainly not ACR. Unsupervised learning methods like cluster or archetypal analysis would avoid the issue of a flawed ground truth at the cost of interpretability. It should be noted that the UCLA external validation cohort was not sampled at random, and samples were selected to ensure relatively balanced representation of stable, infection, and rejection phenotypes. This could introduce bias in performance characteristics. Although promising, significant challenges still exist to bringing this technology to the clinic, including the establishment of benchmark standards, optimization for clinical conditions and demonstration of reproducibility.

We demonstrated that gene expression during ACR is associated with potentially targetable immune responses. Importantly, a genomic classifier can identify lung ACR, including episodes potentially missed by TBB. Late gACR is associated with CLAD, confirming similar clinical importance as late A-grade rejection. BAL-cp gene expression may be a viable alternative to TBB for diagnosing ACR and for stratifying risk of CLAD. These results support a clinical trial where gACR guides use and form of ACR treatment as a strategy to prevent CLAD.

## Contributors

All authors contributed to the acquisition of clinical or biological data for this study. SSW, JZ, TO, MLN, SL, JLT, and JAB contributed to the development of the analysis plan and JZ and TO performed the data analysis. SSW, MLN, and JAB verified the underlying data. All authors contributed to the interpretation of the data. SSW, JZ, TO and SL drafted the manuscript. All authors critically revised the manuscript and approved the final version for submission.

## Data sharing statement

The CTOT-20 protocol has been described in detail.^9^ The full protocol along with deidentified participant data may be made available upon request with written agreement by contacting the corresponding author. Gene expression and clinical data have been deposited Mendeley Data accessed from reserved https://doi.org/10.17632/3x49zhc9r6.1.

## Declaration of interests

S. Sam Weigt has received research grants from CareDx, and Zambon; consulting fees from Boehringer Ingelheim; and honoraria for presentations and speaker's bureau Boehringer Ingelheim and CareDX, and has a provisional patent application entitled Biomarker for allograft injury or rejection.

Jin Zhou has received research grant funding from National Institutes of Health and the National Science Foundation.

Tomoki Okuno has nothing to report.

Megan L. Neely has received research grant funding from the National Institutes of Health and honoraria for lectures in Summer Institute for Biostatistics Program an NC State University.

Shahrzad Lari has nothing to report.

Vyacheslav Palchevskiy has nothing to report.

Jamie L. Todd acknowledges institutional grants from the National Institutes of Health, Sanofi, Avalyn, Boehringer Ingelheim, AstraZeneca, Avalyn, and CareDx, consulting fees from Sanofi, and participation on a data safety monitoring or advisory board for Avalyn, and Sanofi.

Laurie D. Snyder has received research grants from the National Institutes of Health.

David Sayah has received consulting fees from CareDx, speaker's bureau Honoraria from Takeda, and advisory board work for Replimune.

Michael Y. Shino has nothing to report.

John M. Reynolds has nothing to report.

Pali D. Shah has received an institutional research grant from Breath Therapeutics and has participated on a data safety monitoring board or advisory board for the National Institutes of Health.

Lianne G. Singer has received an institutional research grant from Incyte; author royalties from UpToDate, consulting fees from Altavant and Sanofi; and participation on a data safety monitoring or advisory board for Zambon and Renovion.

Marie Budev has nothing to report.

Scott M. Palmer has received research grant funding from Incyte, AstraZeneca, Bristol Myers Squibb, CareDx, and Boehringer Ingelheim; royalties from UptoDate; consulting fees from Altavant Sciences, Bristol Myers Squibb, Boehringer Ingelheim Pharmaceuticals, Mallinckrodt Pharmaceuticals, Abbvie, and Sanofi; and participated on a data safety monitoring board or advisory board for Replimune.

John A. Belperio has nothing to report.

## Appendix A Supplementary data

Figure S1 RNA quantity is associated with volume of BAL fluid saved for research. (A) Samples with sufficient RNA quantity for RNA-Seq were derived from larger volumes of BAL fluid. (B) The proportion of samples failing quality control for insufficient RNA quantity decreases with increasing volumes of BAL fluid submitted for research.

Figure S2 Variable importance for 31 genes included in the random forest model for gACR.

Figure S3 Genomic ACR classifier scores for stable controls (SC) and clinically significant ACR (csACR) events in each surveillance window. In each window, gACR score was significantly higher for csACR than SC (false discovery rate adjusted Mann-Whitney Test).

Figure S4 According to TBBX injury pattern, genomic ACR classifier scores (A) and proportion classified as genomic ACR (B).

Figure S5 According to infection subgroup, ACR classifier scores (A) and proportion classified as ACR (B).

Figure S6 According to AMR subgroup, ACR classifier scores (a) and proportion classified as ACR (b).

Figure S7 Individual plots of genomic ACR classifier score over time for 143 subjects with at least 2 usable samples. Surveillance or for cause indication, as well as A-grade (none, minimal or mild/moderate) are labelled directly on the line. On timelines below graphs there are additional labels for augmented immune suppression, infection, injury (lymphocytic bronchiolitis, organizing pneumonia, or acute lung injury), CLAD (onset, probable, and definite), censor, and death.

Table S1 Group names and definitions.

Table S2 Sample level characteristics of CTOT-20 samples that passes or failed QC.

Table S3 Top 20 Predicted upstream regulators identified by Ingenuity Pathway Analysis (IPA).

Table S4 Performance characteristics for BAL-cp genomic classifier for differentiating clinically significant ACR (csACR) from stable controls (SC) in each surveillance window.

Table S5 Performance Characteristics for BAL-cp Genomic Classifier for differentiating clinically significant ACR from other phenotypes in full CTOT-20 cohort.

Table S6 Cross tabulation of biopsy A-grade and classifier determined genomic ACR.
