# Supplementary figures and images for: Development and validation of a bronchoalveolar lavage genomic classifier for acute cellular rejection
Source: eBioMedicine. 2025 Dec 2;122:106046. doi: 10.1016/j.ebiom.2025.106046 (PMC12719680; doi:10.1016/j.ebiom.2025.106046)

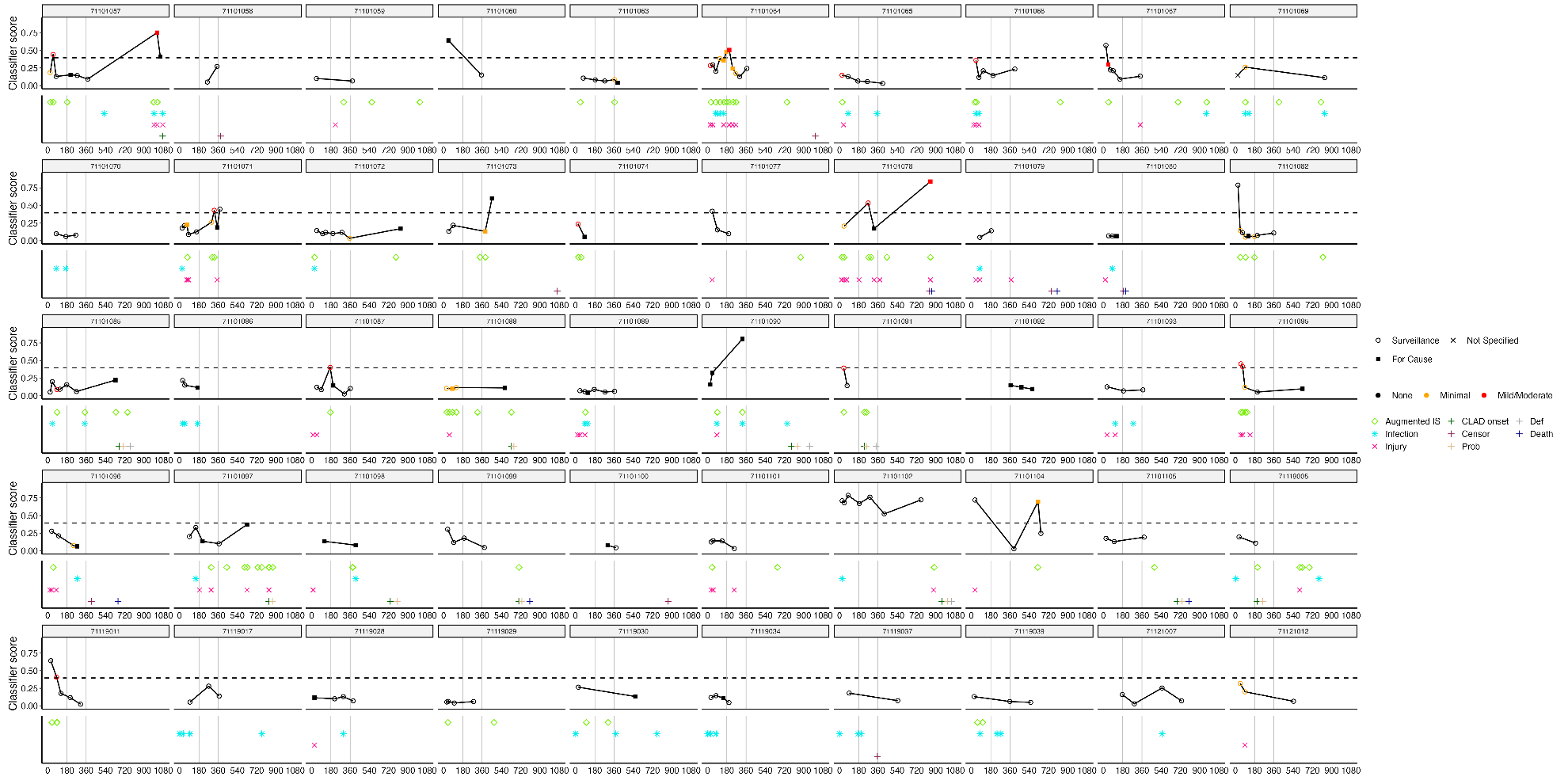


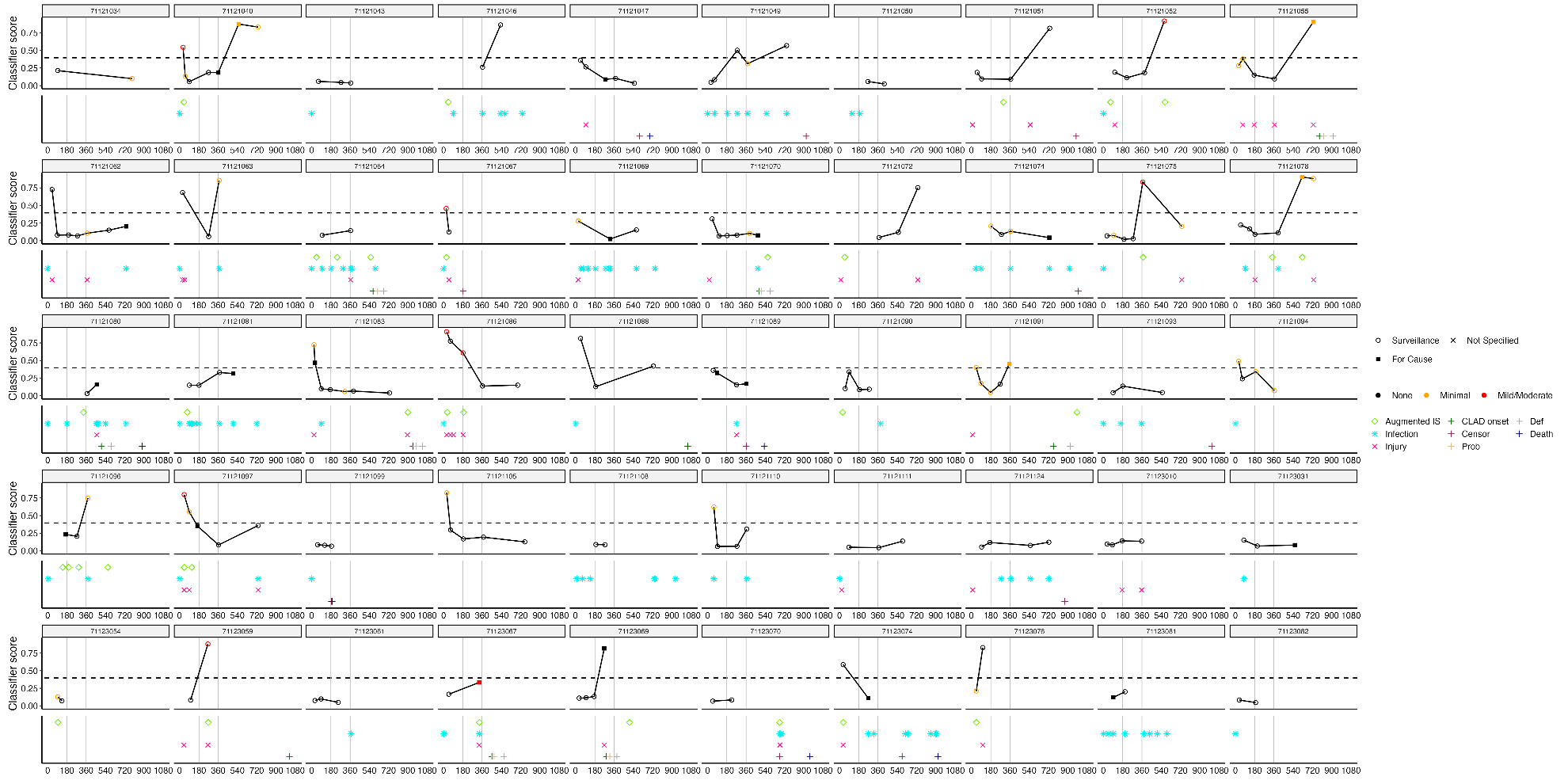


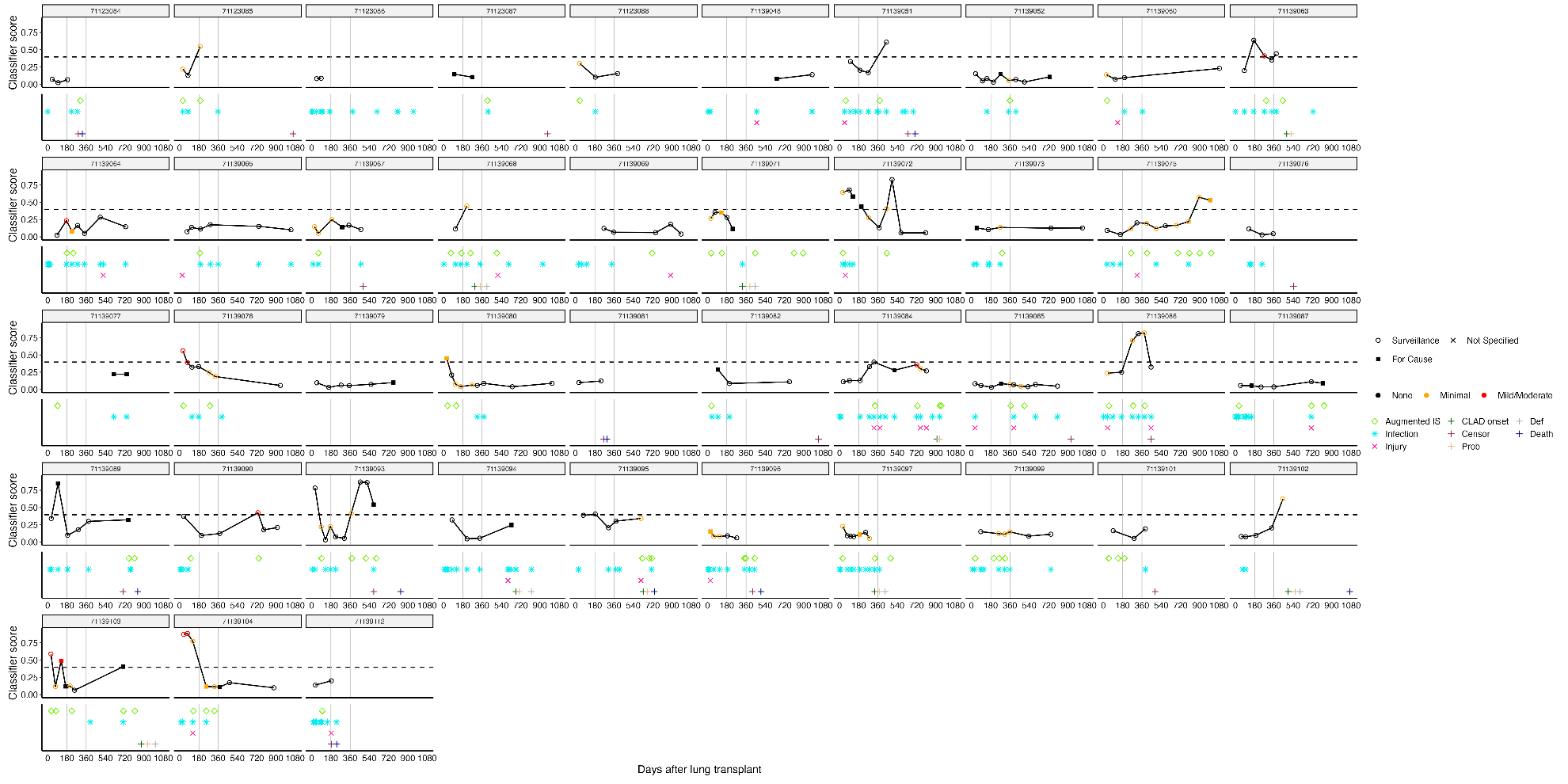

Supplement: Figure S7 — Individual plots of genomic ACR classifier score over time for 143 subjects with at least 2 usable samples. Surveillance or for cause indication, as well as A-grade (none, minimal or mild/moderate) are labelled directly on the line. On timelines below graphs there are additional labels for augmented immune suppression, infection, injury (lymphocytic bronchiolitis, organizing pneumonia, or acute lung injury), CLAD (onset, probable, and definite), censor, and death. [file mmc7.docx]
