# Supplementary material for: Development and validation of a bronchoalveolar lavage genomic classifier for acute cellular rejection
Source: eBioMedicine. 2025 Dec 2;122:106046. doi: 10.1016/j.ebiom.2025.106046 (PMC12719680; doi:10.1016/j.ebiom.2025.106046)
Supplement: Table S1 — Group names and definitions. [file mmc8.docx]

| **Group name** | **Group definition** | **CTOT N (%)** | **UCLA N (%)** |
| --- | --- | --- | --- |
| Stable control (SC) | Surveillance (no allograft dysfunction), no DSA, no pathology (ALI, OP, C4D, or other), no AR, and no infection | 151 (18.7) | 48 (21.9) |
| Clinically significant acute cellular rejection (csACR) | A2 or greater A-grade, or grade A1 plus for cause indication (allograft dysfunction) | 37 (4.6) | 30 (13.7) |
| Asymptomatic A1 (AR1S) | Surveillance and A1 A-grade and no infection. | 41 (5.1) | 31 (14.2) |
| Acute lung allograft dysfunction of unknown etiology (ALAD_UNK) | For cause indication (allograft dysfunction) with negative or ungradable ACR, +/- allograft injury (ALI, OP, LB), and no DSA | 36 (4.5) | 23 (10.5) |
| Allograft injury of unknown etiology (INJ_UNK) | Surveillance (no allograft dysfunction) and positive for allograft injury (ALI, OP, LB), with no ACR, no DSA | 16 (2.0) | 44 (20.1) |
| Probable clinical AMR (AMR_PB_C) | For cause indication (allograft dysfunction), DSA, pathology (ALI, OP, C4D, or other) and no alternative explanation (negative for infection, ACR) | 8 (1.0) | 0 |
| Possible clinical AMR (AMR_Ps_C) | For cause indication (allograft dysfunction), DSA, negative pathology, and no alternate explanation (infection negative, ACR negative) | 34 (4.2) | 1 (0.5) |
| Probable subclinical AMR (AMR_PB_S) | Surveillance (no allograft dysfunction), DSA, pathology (ALI, OP, C4D, or other), and no ACR, no infection | 29 (3.6) | 0 |
| Donor Specific Antibody Possible subclinical AMR (DSA) | Surveillance (no allograft dysfunction), DSA, no pathology (ALI, OP, C4D, or other), no ACR, and no infection | 143 (17.7) | 1 (0.5) |
| Combined csACR and possible AMR (ACR_AMR) | For cause indication (allograft dysfunction), A1 or greater A-grade, DSA, and no infection | 9 (1.1) | 0 |
| Bacterial colonization (INF_BC) | Surveillance (no allograft dysfunction), no DSA, no pathology (ALI, OP, C4D, or other), no ACR, and positive for bacterial pathogen | 42 (5.2) | 13 (5.9) |
| Bacterial pneumonia (INF_BP) | For cause indication (allograft dysfunction), no DSA, no pathology (ALI, OP, C4D, or other), no ACR, and positive for bacterial pathogen | 12 (1.5) | 6 (2.7) |
| CMV pneumonitis (INF_CMV) | Biopsy proven CMV pneumonitis | 1 (0.1) | 0 |
| Fungal colonization (INF_FC) | Surveillance (no allograft dysfunction), no DSA, no pathology (ALI, OP, C4D, or other), no ACR, and positive for mold pathogen | 16 (2.0) | 10 (4.6) |
| Invasive fungal infection (INF_IFI) | For cause indication plus mold pathogen, or biopsy proven IFI | 6 (0.7) | 1 (0.5) |
| Mixed type infection (INF_MIX) | Any indication with multiple classes of pathogens, no ACR | 27 (3.3) | 0 |
| NTM infection (INF_NTM) | Any indication with NTM pathogen, no ACR | 17 (2.1) | 0 |
| Resp viral infection (INF_RV) | Any indication with resp viral pathogen, with or without ACR | 59 (7.3) | 10 (4.6) |
| Combined csACR and Infection (ACR_INF) | csACR (see above) plus positive for infection | 4 (0.5) | 0 |
| Combined Asymptomatic A1 and Infection (AR1S_INF) | Surveillance and A1 A-grade and infectious pathogen (but no infiltrates or purulence). | 13 (1.6) | 0 |
| Combined Asymptomatic A1 and DSA (AR1S_DSA) | Surveillance and A1 A-grade and DSA and negative for infectious pathogen. | 19 (2.4) | 0 |
| Possible subclinical AMR and Infection (DSA_INF) | Surveillance (no allograft dysfunction), DSA, no pathology (ALI, OP, C4D, or other), no ACR, and positive for infectious pathogen | 34 (4.2) | 1 (0.5) |
| Unknown (UNK) | missing indication or missing A-grade (AX) | 52 (6.5) | 0 |
