# Supplementary material for: Development and validation of a bronchoalveolar lavage genomic classifier for acute cellular rejection
Source: eBioMedicine. 2025 Dec 2;122:106046. doi: 10.1016/j.ebiom.2025.106046 (PMC12719680; doi:10.1016/j.ebiom.2025.106046)
Supplement: Table S2 — Sample level characteristics of CTOT-20 samples that passes or failed QC. [file mmc9.docx]

|  | Overall | Low Quantity | Low Integrity | Pass |
| --- | --- | --- | --- | --- |
| **n** | 991 | 148 | 37 | 806 |
| **SITE (%)** |  |  |  |  |
| Cleveland Clinic Foundation | 272 (27.4) | 77 (52.0) | 2 ( 5.4) | 193 (23.9) |
| Duke University Medical Center | 288 (29.1) | 29 (19.6) | 18 (48.6) | 241 (30.0) |
| Johns Hopkins Hospital | 57 ( 5.8) | 6 ( 4.1) | 1 ( 2.7) | 50 ( 6.2) |
| Ronald Reagan UCLA Medical Center | 78 ( 7.9) | 1 ( 0.7) | 14 (37;8) | 63 ( 7.8) |
| Toronto General Hospital | 296 (29.9) | 35 (23.6) | 2 ( 5.4) | 259 (32.1) |
| **LOBE LAVAGED (%)** |  |  |  |  |
| Allograft L lingula | 137 (13.8) | 22 (14.9) | 8 (21.6) | 107 (13.3) |
| Allograft RML | 737 (74.4) | 107 (72.3) | 20 (54.1) | 610 (75.7) |
| Other | 117 (11.8) | 19 (12.8) | 9 (24.3) | 89 (11.0) |
| **BRONCHOSOPY INDICATION** |  |  |  |  |
| For Cause | 205 (20.7) | 35 (23.6) | 17 (45.9) | 153 (19.0) |
| Surveillance | 786(79.3) | 113 (76.4) | 20 (54.1) | 653 (81.0) |
| **AR_Grade (%)** |  |  |  |  |
| None (A0) | 627 (63.3) | 108 (72.9) | 19 (51.4) | 500 (62.0) |
| Minimal (A1) | 131 (13.2) | 13 ( 8.8) | 3 (8.1) | 115 (14.3) |
| Mild (A2) | 49 ( 4.9) | 8 ( 5.4) | 4 (10.8) | 37 ( 4.6) |
| Moderate (A3) | 2 ( 0.2) | 0 ( 0.0) | 1 ( 2.7) | 1 ( 0.1) |
| Ungradable (AX) | 124 (12.5) | 13 ( 8.8) | 3 (8.1) | 108 (13.4) |
| No Biopsy | 58 ( 5.9) | 6 ( 4.1) | 7 (18.9) | 45 ( 5.6) |
| **LB_Grade (%)** |  |  |  |  |
| None (B0) | 577 (58.2) | 104 (70.3) | 16 (43.2) | 457 (56.7) |
| Low-grade lymphocytic bronchiolitis (B1R) | 28 ( 2.8) | 6 ( 4.1) | 3 ( 8.1) | 19 ( 2.4) |
| High-grade lymphocytic bronchiolitis (B2R) | 1 ( 0.1) | 1 ( 0.7) | 0 ( 0.0) | 0 ( 0.0) |
| Ungradable (BX) | 327 (33.0) | 31 (20.9) | 11 (29.7) | 285 (35.4) |
| No Biopsy | 58 ( 5.9) | 6 ( 4.1) | 7 (18.9) | 45 ( 5.6) |
| **ALI (%)** |  |  |  |  |
| No ALI | 890 (89.8) | 138 (93.2) | 28 (75.7) | 724 (89.8) |
| ALI Present | 43 ( 4.3) | 4 ( 2.7) | 2 ( 5.4) | 37 ( 4.6) |
| No Biopsy | 58 ( 5.9) | 6 ( 4.1) | 7 (18.9) | 45 ( 5.6) |
| **OP (%)** |  |  |  |  |
| No OP | 890 (89.8) | 137 (92.6) | 29 (78.4) | 724 (89.8) |
| OP Present | 43 ( 4.3) | 5 ( 3.4) | 1 ( 2.7) | 37 ( 4.6) |
| No Biopsy | 58 ( 5.9) | 6 ( 4.1) | 7 (18.9) | 45 ( 5.6) |
| **BACTPOS (%)** |  |  |  |  |
| No | 837 (84.5) | 130 (87.8) | 27 (72.9) | 680 (84.4) |
| Yes | 146 (14.7) | 18 (12.2) | 9 (24.3) | 119 (14.8) |
| Not tested | 8 ( 0.8) | 0 ( 0.0) | 1 ( 2.7) | 7 ( 0.9) |
| **MYCOPOS (%)** |  |  |  |  |
| No | 934 (94.2) | 142 (95.9) | 32 (86.5) | 760 (94.3) |
| Yes | 44 ( 4.4) | 4 ( 2.7) | 5 (13.5) | 35 ( 4.3) |
| Not tested | 13 ( 1.3) | 2 ( 1.4) | 0 ( 0.0) | 11 ( 1.4) |
| **FUNGPOS (%)** |  |  |  |  |
| No | 781 (78.8) | 130 (87.8) | 24 (64.9) | 627 (77.8) |
| Yes | 208 (21.0) | 18 (12.2) | 13 (35.1) | 177 (22.0) |
| Not tested | 2 ( 0.2) | 0 ( 0.0) | 0 ( 0.0) | 2 ( 0.2) |
| **VIRLPOS (%)** |  |  |  |  |
| No | 877 (88.5) | 133 (89.9) | 30 (81.1) | 714 (88.6) |
| Yes | 102 (10.3) | 14 ( 9.5) | 7 (18.9) | 81 (10.0) |
| Not tested | 12 ( 1.2) | 1 ( 0.7) | 0 ( 0.0) | 11 ( 1.4) |
| **BAL VOL ml INST, mean (SD)** | 85.6 (23.5) | 79.4 (19.3) | 104.1 (28.0) | 85.8 (23.6) |
| **BAL VOL ml RETR, mean (SD)** | 41.9 (11.1) | 39.6 (9.9) | 44.1 (14.1) | 42.2 (11.1) |
| **BAL ml SUBMITTED TO BIOBANK, mean (SD)** | 16.2 (11.3) | 9.4 (7.1) | 23.3 (12.3) | 17.1 (11.4) |
| **DAYS POST-TRANSPLANT TO BAL, mean (SD)** | 278.6 (234.5) | 280.1 (226.8) | 298.0 (285.1) | 277.5 (233.7) |
