# Supplementary material for: Development and validation of a bronchoalveolar lavage genomic classifier for acute cellular rejection
Source: eBioMedicine. 2025 Dec 2;122:106046. doi: 10.1016/j.ebiom.2025.106046 (PMC12719680; doi:10.1016/j.ebiom.2025.106046)
Supplement: Table S3 — Top 20 Predicted upstream regulators identified by Ingenuity Pathway Analysis (IPA). [file mmc10.docx]

| **Upstream Regulator** | **Molecule Type** | **Activation z-score** | **p-value of overlap** | **Target Molecules in Dataset** |
| --- | --- | --- | --- | --- |
| IL2 | cytokine | 3.631 | 2.2E-25 | CCL5, CD2, CD247, CD8A, CD8B, CST7, CTLA4, CXCL9, CXCR3, CXCR6, ETS1, GZMA, GZMB, GZMH, GZMK, IKZF3, IL2RB, ITK, KLRD1, LCK, LINC02446, PDCD1, PRF1, PTPN7, SIRPG, TIGIT |
| pembrolizumab | biologic drug | 3.317 | 1.11E-22 | CCL5, CD27, CD8A, CTLA4, CXCL9, CXCR6, GZMA, GZMB, LAG3, PDCD1, PRF1, TIGIT |
| CD3 (complex) | complex | 2.076 | 1.46E-21 | CCL5, CD2, CD247, CD27, CD3E, CD8A, CD8B, CTLA4, CXCR3, CXCR6, ETS1, GZMA, GZMB, GZMK, IL2RB, IL32, LCK, PDCD1, PRF1, PTPN7, SH2D2A, TIGIT |
| IL15 | cytokine | 3.662 | 8.02E-18 | CCL5, CD2, CD8A, CD8B, CD96, CXCR6, ETS1, GNLY, GZMB, GZMH, GZMK, IL2RB, IL32, KLRD1, LCK, NKG7, PDCD1, PRF1 |
| alefacept | biologic drug | -2.97 | 1.63E-17 | CCL5, CD2, CD8A, CST7, CXCL9, GNLY, GZMB, IL2RB, KLRD1, PRF1 |
| EP4-D | chemical reagent | -3.162 | 3.48E-17 | CCL5, CD27, CD3E, CXCR3, GZMA, GZMB, IKZF3, IL2RB, LCK, PRF1 |
| CUL5 | ion channel | -2.345 | 1.85E-16 | CCL5, CD247, CD3E, CTLA4, IL2RB, LAG3, PDCD1, PRF1, TIGIT |
| IL18 | cytokine | 2.59 | 3.69E-16 | CCL5, CD96, CXCL9, GZMA, GZMB, GZMH, GZMK, GZMM, IL32, NKG7, PDCD1, PRF1, TIGIT |
| IL12 (family) | group | 3.369 | 5.6E-16 | CCL5, CD3E, CD8A, CD96, CXCR6, GZMB, GZMH, IKZF3, KLRD1, NKG7, PDCD1, PRF1 |
| TBX21 | transcription regulator | 1.526 | 9.97E-16 | CCL5, CXCL9, CXCR3, GZMA, GZMB, IL2RB, ITK, LAG3, NKG7, PDCD1, PRF1 |
| KLRK1 | transmembrane receptor | 1.701 | 1.71E-15 | CCL5, CD96, GZMA, GZMB, LAG3, NKG7, PRF1, SLA2, TIGIT |
| IL21 | cytokine | 3.06 | 3.03E-15 | CCL5, CD2, CD27, CXCL13, CXCL9, CXCR3, GZMA, GZMB, IL2RB, KLRD1, PDCD1, PRF1 |
| FOXP3 | transcription regulator | 1 | 4.74E-15 | CCL5, CTLA4, CXCL13, CXCR6, GZMA, GZMB, GZMK, IL2RB, LAG3, PDCD1, PRF1, TIGIT |
| EBF4 | transcription regulator | 2.443 | 1.22E-14 | GZMA, GZMK, IL2RB, KLRD1, NKG7, PRF1 |
| TCR (complex) | complex | 2.375 | 3.58E-14 | CCL5, CD27, CD8B, CTLA4, CXCL13, CXCL9, CXCR3, GZMA, GZMB, GZMH, LCK, PDCD1, PRF1, ZAP70 |
| TCF7 | transcription regulator | 0.862 | 6.04E-14 | CCL5, CD27, CD3E, CTLA4, CXCR3, GZMA, GZMB, IKZF3, IL2RB, LAG3, LCK, PDCD1, PRF1, TIGIT |
| NFAT5 | transcription regulator | -3.583 | 9.21E-13 | CCL5, CD247, CD27, CD7, CD8A, CD8B, CTLA4, CXCL9, CXCR3, CXCR6, GZMA, LCK, NKG7, ZAP70 |
| STAT3 | transcription regulator | 1.611 | 1.14E-12 | CCL5, CTLA4, CXCL13, CXCL9, CXCR3, CXCR6, ETS1, GZMA, GZMB, GZMH, GZMK, IKZF3, IL2RB, LAG3, PDCD1, PRF1, TIGIT |
| semaxinib | chemical drug |  | 1.56E-12 | CD247, CD3E, CD8A, CD8B, CXCL13, CXCL9, IL2RB, ITK, LCK, ZAP70 |
| ETS1 | transcription regulator | -0.174 | 2.95E-12 | CCL5, CD27, ETS1, GZMA, GZMB, GZMK, IL2RB, ITK, LCK, PRF1, ZAP70 |
