# Supplementary material for: Development and validation of a bronchoalveolar lavage genomic classifier for acute cellular rejection
Source: eBioMedicine. 2025 Dec 2;122:106046. doi: 10.1016/j.ebiom.2025.106046 (PMC12719680; doi:10.1016/j.ebiom.2025.106046)
Supplement: Table S4 — Performance characteristics for BAL-cp genomic classifier for differentiating clinically significant ACR (csACR) from stable controls (SC) in each surveillance window. [file mmc11.docx]

|  | **1-month** | **3-month** | **6-month** | **9-month** | **1 year** | **>1 year** |
| --- | --- | --- | --- | --- | --- | --- |
| **csACR (n)** | 15 | 6 | 5 | 3 | 3 | 5 |
| **SC (n)** | 28 | 34 | 25 | 22 | 17 | 26 |
| **sensitivity** | 66.7% | 66.7% | 100.0% | 66.7% | 100.0% | 100.0% |
| **specificity** | 89.3% | 100% | 96.0% | 100.0% | 100.0% | 88.5% |
| **accuracy** | 81.4% | 95% | 96.7% | 96.0% | 100.0% | 90.3% |
