# Supplementary material for: Development and validation of a bronchoalveolar lavage genomic classifier for acute cellular rejection
Source: eBioMedicine. 2025 Dec 2;122:106046. doi: 10.1016/j.ebiom.2025.106046 (PMC12719680; doi:10.1016/j.ebiom.2025.106046)
Supplement: Table S5 — Performance Characteristics for BAL-cp Genomic Classifier for differentiating clinically significant ACR from other phenotypes in full CTOT-20 cohort. [file mmc12.docx]

| Cohort |  | ACR definition | Sensitivity | Specificity | PPV | NPV | Accuracy | Prevalence |
| --- | --- | --- | --- | --- | --- | --- | --- | --- |
| Cases and controls |  | csACR vs. SC | 78.4% | 95.4% | - | - | 92.0% | - |
| All CTOT-20 samples |  | A1 or greater vs others | 36.0% | 89.9% | 53.4% | 81.3% | 76.7% | 24.4% |
|  |  | A2 or greater vs others | 71.1% | 87.6% | 26.2% | 98.0% | 86.7% | 5.8% |
|  |  | A1 or B1R or greater vs others | 32.3% | 91.0% | 56.3% | 78.8% | 75.4% | 26.6% |
| CTOT-20 samples excluding infection |  | A1 or greater vs others | 40.6% | 90.9% | 59.1% | 82.6% | 78.7% | 24.4% |
|  |  | A2 or greater vs others | 75.0% | 87.7% | 31.8% | 97.9% | 86.8% | 7.1% |
|  |  | A1 or B1R or greater vs others | 37.5% | 92.1% | 63.8% | 79.8% | 77.3% | 27.1% |
| CTOT-20 surveillance samples |  | A1 or greater vs others | 32.8% | 91.6% | 54.6% | 81.5% | 77.7% | 23.6% |
|  |  | A2 or greater vs others | 71.0% | 89.2% | 28.6% | 98.1% | 88.2% | 5.7% |
|  |  | A1 or B1R or greater vs others | 30.5% | 92.5% | 60.4% | 78.0% | 75.6% | 27.3% |
| CTOT-20 surveillance samples excluding infection |  | A1 or greater vs others | 37.0% | 91.4% | 57.7% | 82.2% | 78.4% | 24.0% |
|  |  | A2 or greater vs others | 76.0% | 89.5% | 36.5% | 97.9% | 88.5% | 7.2% |
|  |  | A1 or B1R or greater vs others | 35.3% | 92.3% | 63.2% | 79.2% | 76.7% | 27.3% |
| CTOT-20 for cause samples |  | A1 or greater vs others | 52.0% | 84.7% | 50.0% | 85.7% | 77.3% | 22.7% |
|  |  | A2 or greater vs others | 71.4% | 79.6% | 19.2% | 97.6% | 79.1% | 6.4% |
|  |  | A1 or B1R or greater vs others | 42.1% | 83.9% | 44.4% | 82.5% | 74.1% | 23.5% |
| CTOT-20 for cause samples excluding infection |  | A1 or greater vs others | 60.0% | 87.5% | 64.3% | 85.4% | 80% | 27.3% |
|  |  | A2 or greater vs others | 66.7% | 76.9% | 14.3% | 97.6% | 76.4% | 5.5% |
|  |  | A1 or B1R or greater vs others | 50.0% | 90.9% | 66.7% | 83.3% | 89.0% | 26.7% |
