# Supplementary material for: Development and validation of a bronchoalveolar lavage genomic classifier for acute cellular rejection
Source: eBioMedicine. 2025 Dec 2;122:106046. doi: 10.1016/j.ebiom.2025.106046 (PMC12719680; doi:10.1016/j.ebiom.2025.106046)
Supplement: Table S6 — Cross tabulation of biopsy A-grade and classifier determined genomic ACR. [file mmc13.docx]

|  | **Samples with Adequate BAL-cp RNA** | | | | | |
| --- | --- | --- | --- | --- | --- | --- |
|  | **Total** | **A0** | **A1** | **≥A2** | **AX** | **No biopsy** |
|  | 806 | 500 | 115 | 38 | 108 | 45 |
| **gACR+** | 138 (17.1%) | 48 (9.6%) | 28 (24.3%) | 27 (71.1%) | 22 (20.4%) | 13 (28.9%) |
| **gACR-** | 668 (82.9%) | 452 (90.4%) | 87 (73.7%) | 11 (28.9%) | 86 (79.6%) | 32 (71.1%) |
