# Supplementary material for: Development and validation of a bronchoalveolar lavage genomic classifier for acute cellular rejection
Source: eBioMedicine. 2025 Dec 2;122:106046. doi: 10.1016/j.ebiom.2025.106046 (PMC12719680; doi:10.1016/j.ebiom.2025.106046)
Supplement: Supplementary file 8 [file mmc1.docx]

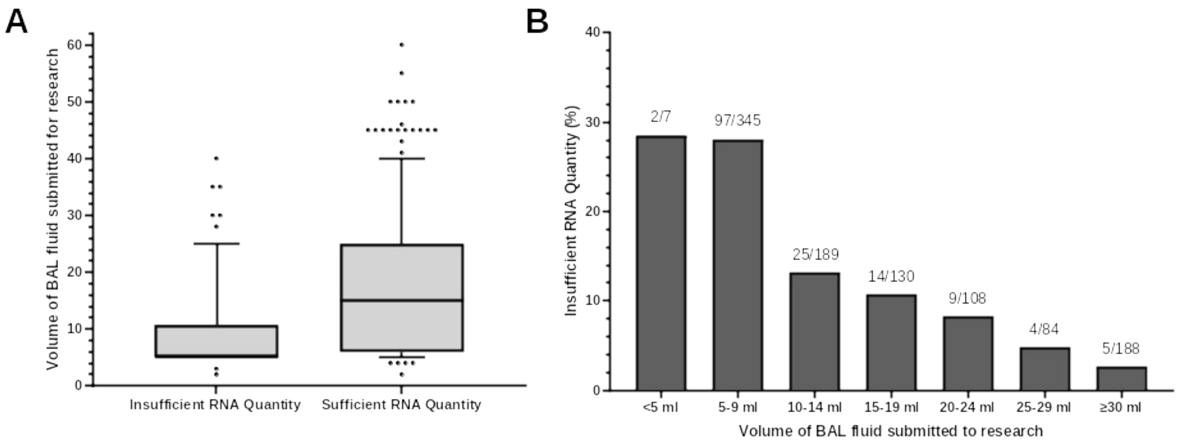
A
Volume of BAL fluid submitted for research
0
10
20
30
40
50
60
Insufficient RNA Quantity
Sufficient RNA Quantity
B
Insufficient RNA Quantity (%)
0
10
20
30
40
2/7
97/345
25/189
14/130
9/108
4/84
5/188
<5 ml
5-9 ml
10-14 ml
15-19 ml
20-24 ml
25-29 ml
≥30 ml
Volume of BAL fluid submitted to research
