# Supplementary material for: Development and validation of a bronchoalveolar lavage genomic classifier for acute cellular rejection
Source: eBioMedicine. 2025 Dec 2;122:106046. doi: 10.1016/j.ebiom.2025.106046 (PMC12719680; doi:10.1016/j.ebiom.2025.106046)
Supplement: Supplementary file 10 [file mmc3.docx]

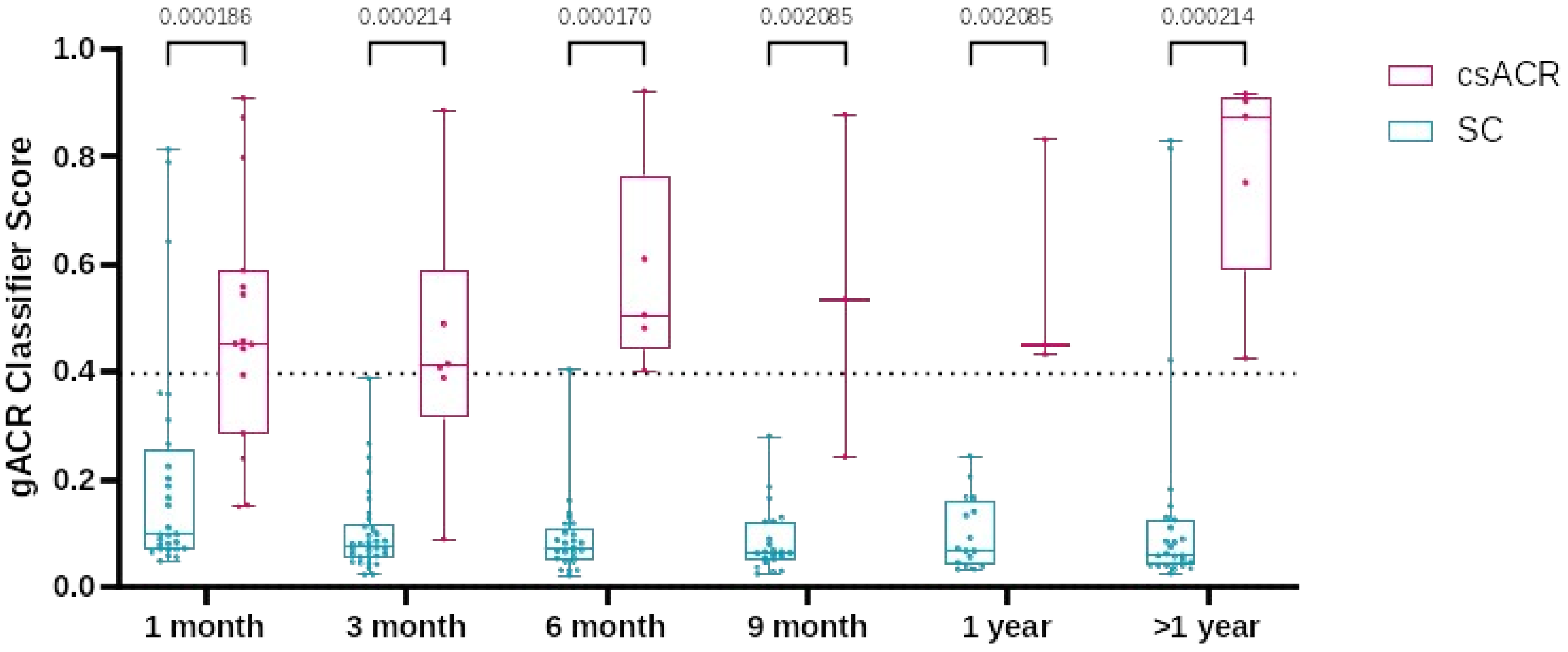
0.000186
0.000214
0.000170
0.002085
0.002085
0.000214
1.0
0.8
0.6
0.4
0.2
0.0
gACR Classifier Score
1 month
3 month
6 month
9 month
1 year
>1 year
csACR
SC
