# Supplementary material for: Development and validation of a bronchoalveolar lavage genomic classifier for acute cellular rejection
Source: eBioMedicine. 2025 Dec 2;122:106046. doi: 10.1016/j.ebiom.2025.106046 (PMC12719680; doi:10.1016/j.ebiom.2025.106046)
Supplement: Supplementary file 12 [file mmc5.docx]

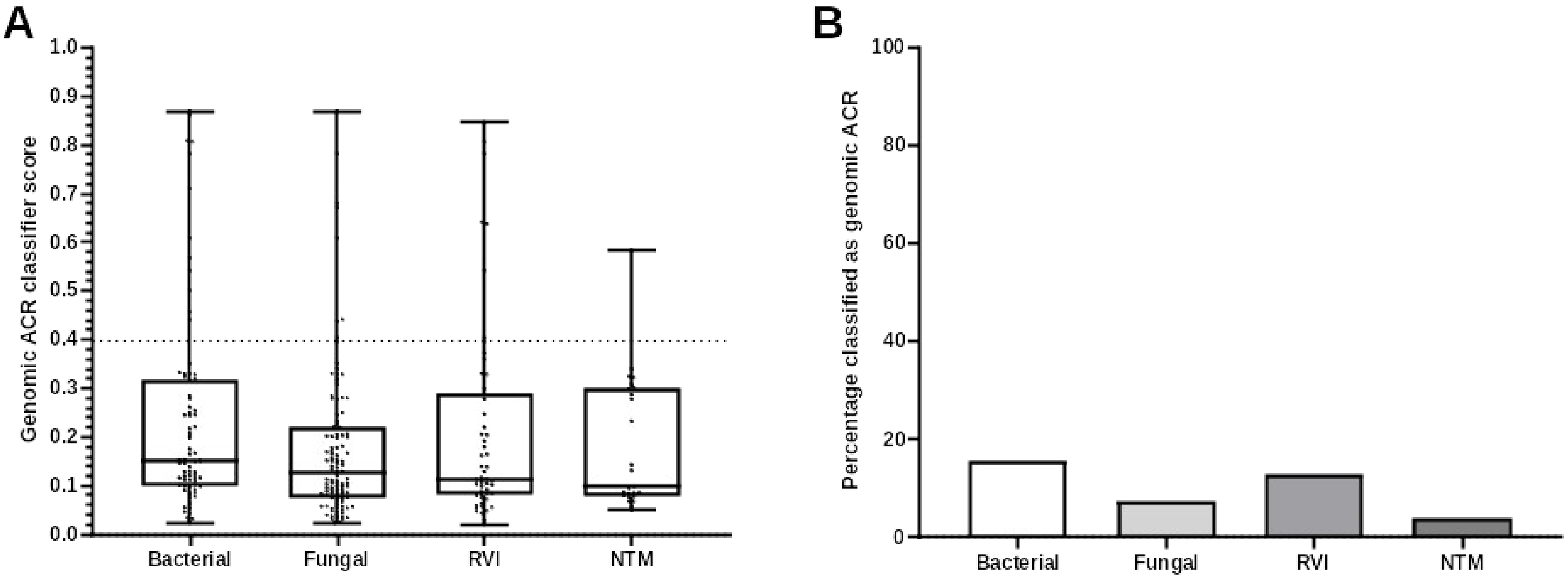
A
Genomic ACR classifier score
1.0
0.9
0.8
0.7
0.6
0.5
0.4
0.3
0.2
0.1
0.0
Bacterial
Fungal
RVI
NTM
B
Percentage classified as genomic ACR
100
80
60
40
20
0
Bacterial
Fungal
RVI
NTM
