# Supplementary material for: Development and validation of a bronchoalveolar lavage genomic classifier for acute cellular rejection
Source: eBioMedicine. 2025 Dec 2;122:106046. doi: 10.1016/j.ebiom.2025.106046 (PMC12719680; doi:10.1016/j.ebiom.2025.106046)
Supplement: Supplementary file 13 [file mmc6.docx]

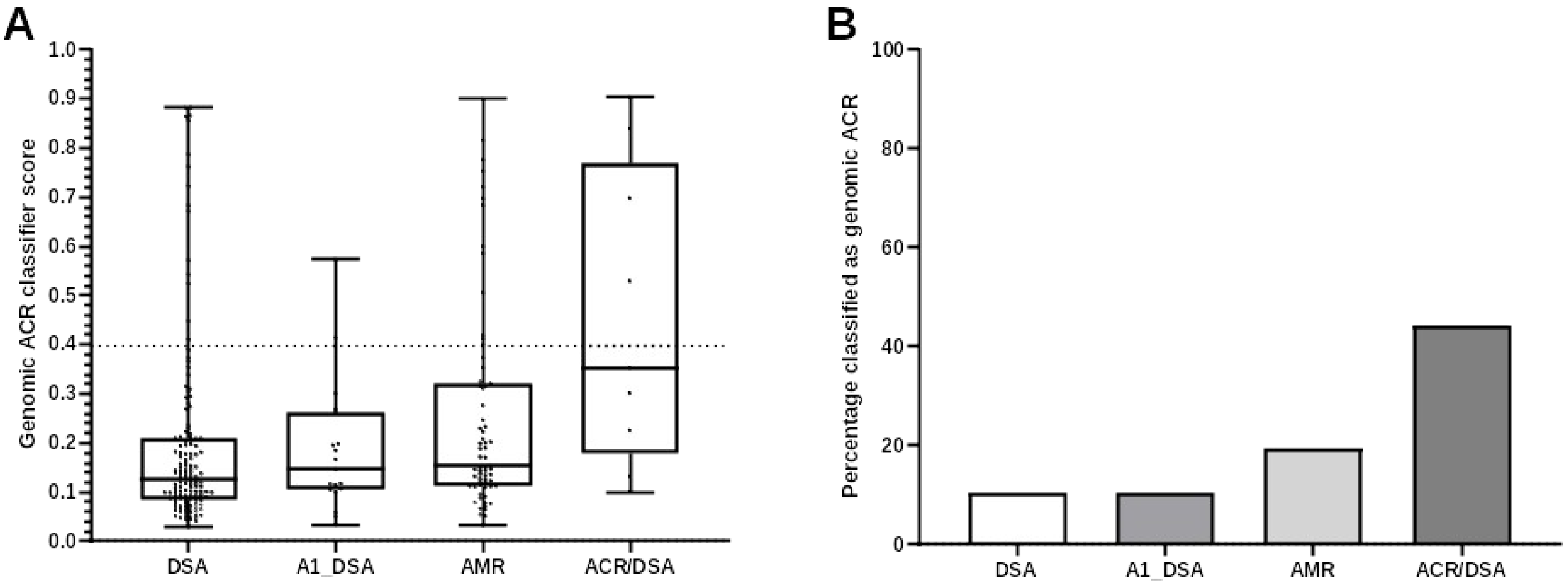
A
1.0
0.9
0.8
0.7
0.6
0.5
0.4
0.3
0.2
0.1
0.0
Genomic ACR classifier score
DSA
A1_DSA
AMR
ACR/DSA
B
100
80
60
40
20
0
Percentage classified as genomic ACR
DSA
A1_DSA
AMR
ACR/DSA
